# Model-based analysis of response and resistance factors of cetuximab treatment in gastric cancer cell lines

**DOI:** 10.1371/journal.pcbi.1007147

**Published:** 2020-03-02

**Authors:** Elba Raimúndez, Simone Keller, Gwen Zwingenberger, Karolin Ebert, Sabine Hug, Fabian J. Theis, Dieter Maier, Birgit Luber, Jan Hasenauer

**Affiliations:** 1 Helmholtz Zentrum München-German Research Center for Environmental Health, Institute of Computational Biology, Neuherberg, Germany; 2 Center for Mathematics, Technische Universität München, Garching, Germany; 3 Technical University of Munich, School of Medicine, Klinikum rechts der Isar, Institute of Pathology, Munich, Germany; 4 Biomax Informatics AG, Planegg, Germany; 5 Faculty of Mathematics and Natural Sciences, University of Bonn, Bonn, Germany; University at Buffalo - The State University of New York, UNITED STATES

## Abstract

Targeted cancer therapies are powerful alternatives to chemotherapies or can be used complementary to these. Yet, the response to targeted treatments depends on a variety of factors, including mutations and expression levels, and therefore their outcome is difficult to predict. Here, we develop a mechanistic model of gastric cancer to study response and resistance factors for cetuximab treatment. The model captures the EGFR, ERK and AKT signaling pathways in two gastric cancer cell lines with different mutation patterns. We train the model using a comprehensive selection of time and dose response measurements, and provide an assessment of parameter and prediction uncertainties. We demonstrate that the proposed model facilitates the identification of causal differences between the cell lines. Furthermore, our study shows that the model provides predictions for the responses to different perturbations, such as knockdown and knockout experiments. Among other results, the model predicted the effect of MET mutations on cetuximab sensitivity. These predictive capabilities render the model a basis for the assessment of gastric cancer signaling and possibly for the development and discovery of predictive biomarkers.

## Author summary

Unraveling the causal differences between drug responders and non-responders is an important challenge. The information can help to understand molecular mechanisms and to guide the selection and design of targeted therapies. Here, we approach this problem for cetuximab treatment for gastric cancer using mechanistic mathematical modeling. The proposed model describes responder and non-responder gastric cancer cell lines and can predict the response in several validation experiments. Our analysis provides a differentiated view on mutations and explains, for instance, the relevance of MET mutations and the insignificance of PIK3CA mutation in the considered cell lines. The model might potentially provide the basis for understanding the recent failure of several clinical studies.

## Introduction

Gastric cancer is the fifth most common cancer and third leading cause of death from cancer worldwide [1]. Treatment options include surgery, chemo- and radiation therapy. However, the overall survival rate remains unsatisfactory due to molecular and clinical heterogeneity [2], therefore new treatment options are urgently required. Novel drugs targeting members of a family of receptor tyrosine kinases including the epidermal growth factor receptor (EGFR) have shown mixed success in clinical trials [3, 4]. Among others, the EGFR antibody cetuximab did not improve patient survival in a phase III clinical trial [4].

Cetuximab is an antibody which binds EGFR [5]. EGFR is overepressed in many cancer types and activated by a variate of ligands [6], including besides EGF also the transforming growth factor-alpha (TGFA), heparin-binding EGF-like growth factor (HBEGF), betacellulin (BTC), amphiregulin (AREG) and epiregulin (EREG) and epigen (EPGN). Although all these ligands bind to EGFR in the abscence of cetuximab, they do not produce identical biological responses. These varying responses might be due to different ligand affinity, different ability to induce EGFR recycling or different ability to produce EGFR:ERBB2 heterodimers or EGFR:EGFR homodimers [7]. The binding of cetuximab to EGFR inhibits the interaction with its ligands, thereby reducing survival and proliferation signals. In addition, cetuximab induces antibody-dependent cellular cytotoxicity (ADCC) by provoking immune cells to attack cancer cells [8].

A potential reason for the failure of cetuximab is the molecular heterogeneity of gastric cancer [2]. Due to this heterogeneity, only a small subgroup of patients might benefit from the targeted therapy. However, a suitable biomarker for patient stratification is currently not available. Here, we aim to render a basis for understanding response and resistance mechanisms for cetuximab treatment in gastric cancer, to unravel likely causal differences between cetuximab responders and non-responders [9], and to possibly identify biomarkers for guiding targeted therapy by using a cell culture model.

Conceptually, biomarkers for responsive patient subgroups can be identified using statistical approaches characterizing responder and non-responder subgroups within different molecular high-throughput methods. Unfortunately, the necessary large-scale studies on the response of gastric cancer patients to cetuximab are missing. In addition, many proposed biomarkers from purely associative approaches have failed in clinical use [10]. Even large-scale cancer cell line projects, such as the Cancer Cell Line Encyclopedia (CCLE) [11] and the Genomics of Drug Sensitivity in Cancer (GDSC) [12] project, do not provide data for cetuximab response. Consequently, the limited amount of cell line and patient data prohibits the use of established statistical methods for biomarker development for cetuximab responsive patient subgroups.

In recent years, several studies showed that mechanistic dynamical models provide an alternative route to biomarker development [13]. [14] predicted the survival of neuroblastoma patients using a mechanistic model of the c-Jun N-terminal kinase (JNK) pathway. [6] predicted ligand dependence of solid tumors using a mechanistic multi-pathway model. [15] showed that large-scale mechanistic models facilitate the integration of large-scale data and enable the derivation and mechanistic interpretation of biomarkers. All these studies employ ordinary differential equation (ODE) models to describe the biochemical reaction networks involved in intracellular signal processing. The models integrate (i) prior knowledge on the pathway topology derived over the last decades and available in databases, such as KEGG [16], Reactome [17] and BioModels [18], with (ii) heterogeneous experimental data for the process of interest. The exploitation of prior knowledge constrains the search space and improves in many cases the predictive power. Furthermore, the chosen modeling approach facilitates, unlike most statistical approaches, the mechanistic interpretation of the findings.

In this study, we employed mechanistic mathematical modeling based on ODEs to understand response and resistance mechanisms for cetuximab treatment in gastric cancer cell lines. Building on previously published mechanistic ODE models [6, 15, 19–21] and published logical models [22], we developed multiple candidate models for the EGFR, the Protein Kinase B (AKT) and the Extracellular-signal Regulated Kinase (ERK) signaling in cetuximab responder and non-responder cell lines. The most appropriate model based on the Akaike Information Criterion (AIC) [23] was selected, calibrated and validated using previous published and newly collected data. To analyze the dependence of the cellular response on gene expression levels and (somatic) mutations, the resulting model was interrogated using simulation studies and *in silico* knockdown and knockout experiments. This suggests several intervention points and potential model-based biomarkers.

## Results

### Mathematical model of intracellular signaling in cetuximab responder and non-responder cell lines

We utilized the gastric cancer cell lines MKN1 and Hs746T as a model system to study response and resistance factors of cetuximab treatment. Based on previous results obtained by proliferation and motility analysis, the MKN1 cell line is characterized as a cetuximab responder, while Hs746T is characterized as a non-responder cell line [9, 24]. Different factors might contribute to this difference, including a 2.5 fold higher EGFR expression in MKN1 cells compared to Hs746T [24].

Furthermore, MKN1 cells expresses the PI3K mutant PI3K p.E545K and in Hs746T cells MET is mutated to MET p.L982_D1028del and amplified [25]. PI3K p.E545K possesses an increased catalytic activity compared to wild-type PI3K [26] resulting in enhanced downstream signaling, while MET p.L982_D1028de is assumed to be constitutively active, i.e. independent of its ligand.

Cetuximab targets the EGFR signaling pathway which regulates growth, survival, proliferation, differentiation [27] and motility [9, 28]. Upon ligand binding, EGFR homodimerizes and auto-phosphorylates promoting its catalytic activity. Phosphorylated EGFR (pEGFR) is internalized and degraded or recycled. Membrane-bound and internalized pEGFR catalyzes the activation of the G-protein RAS and the Phosphoinositid-3-Kinase (PI3K) by phosphorylation. While RAS activates the mitogen-activated (MAPK) signaling pathway resulting in the phosphorylation of ERK, PI3K activity results in the phosphorylation of AKT. Phosphorylated ERK (pERK) and phosphorylated AKT (pAKT) are important regulators of DNA transcription.

Cetuximab binds EGFR and blocks the binding of EGF or other EGFR ligands, such as amphiregulin (AREG) and epiregulin (EREG) [5]. This reduces—in the absence of resistance factors—the activity of EGFR and its downstream targets. Known resistance factors to EGFR-targeted therapies in other cancer types include mutations and overexpression of the receptor tyrosine kinases AXL [29] or MET [30].

**Fig 1:**
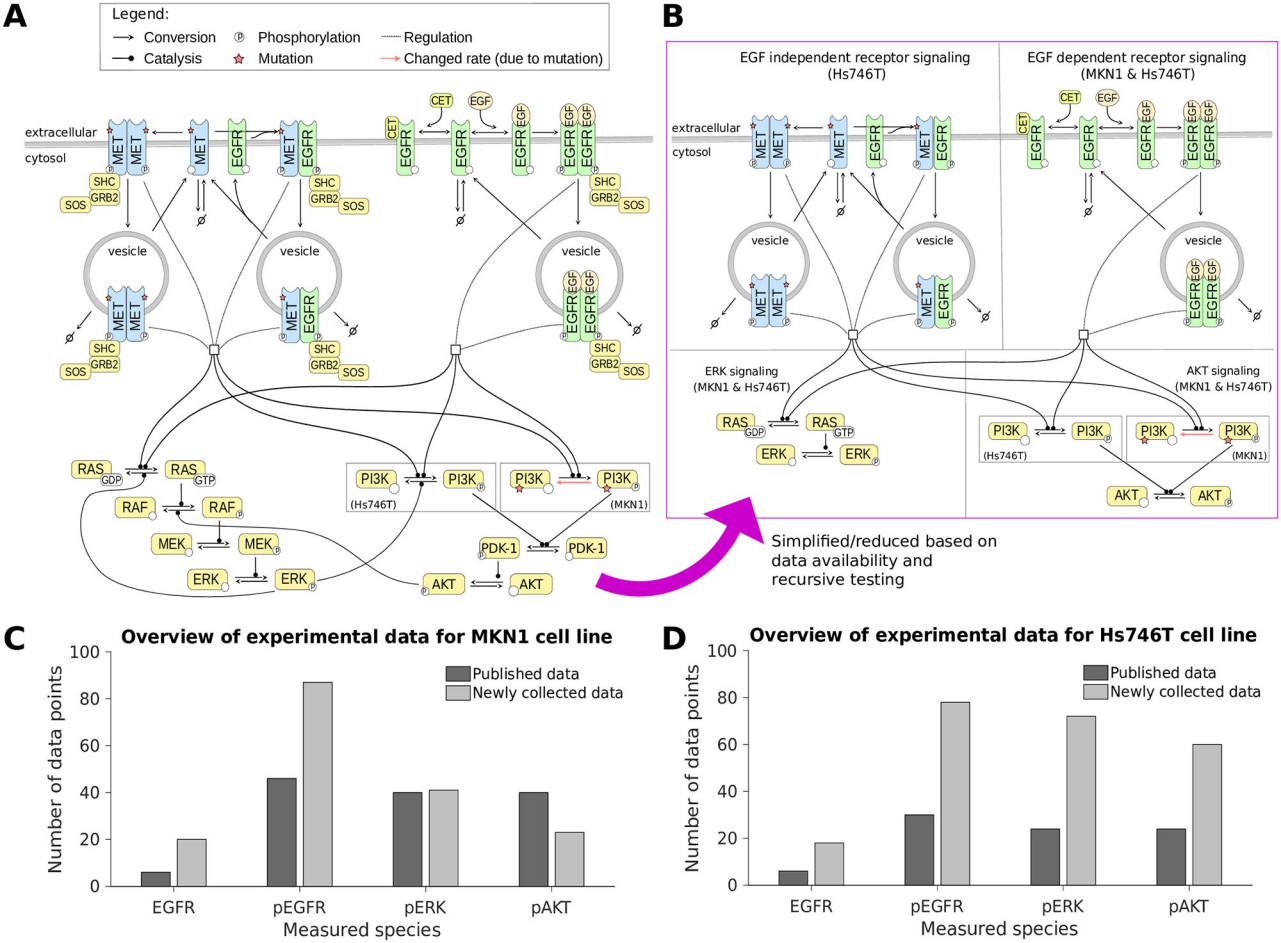
The pathway model and experimental data. A: Illustration of the EGFR/MET signaling pathway, focusing on the ERK and AKT signaling cascades, used to build the mathematical model. B: Illustration of the mathematical model indicating shared and cell-line specific biochemical species/reactions. C-D: Overview of the experimental data used for model calibration for MKN1 and Hs746T cell lines. The amount of data from the literature (published by [9]) and newly collected data (published as part of this study) is distinguished.

A visualization of the EGFR signalling pathway accounting for common adaptor proteins, cetuximab as well as the aforementioned mutants of PI3K and MET is provided in Fig 1A. Based on this pathway information and the available literature, in particular the work of [19] and [21], we constructed a simplified pathway model. To limit the model complexity, adaptor proteins and multi-site phosphorylation of EGFR and ERK are disregarded, and the intermediate steps in the MAPK signaling pathway are lumped into a single reaction. The simplified pathway description is visualized in Fig 1B. The model description is provided in the S1 Text, Section 3.

We calibrated the pathway model using a comprehensive dataset obtained using quantitative immunoblotting. The dataset contains time and dose responses for EGFR, pEGFR, pERK and pAKT. In an iterative process, published data [9, 24] were complemented by newly collected data to improve reliability of the parameter estimates and model predictions. In total, we considered 303 data points for the MKN1 cell line (Fig 1C) and 312 data points for the Hs746T cell line (Fig 1D).

**Fig 2:**
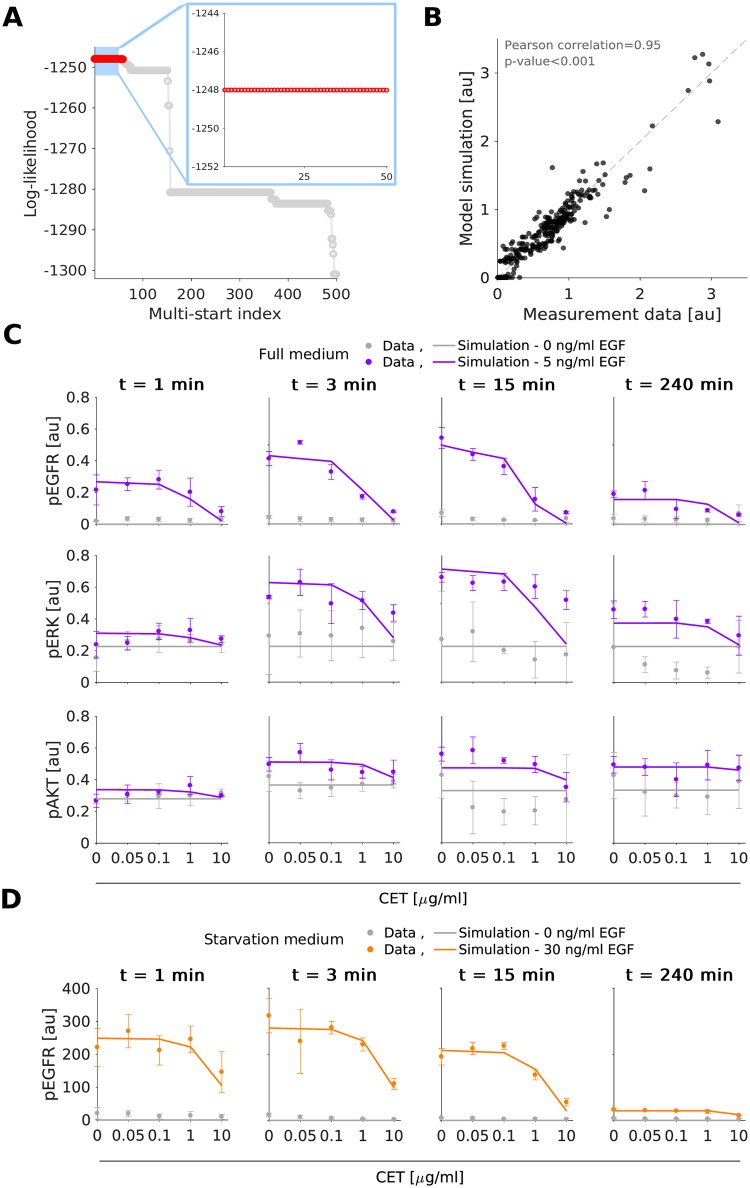
Experimental data and model fit for the gastric cancer cell line MKN1. A: Waterfall plot for multi-start local optimization. The best 500 out of 1000 runs are depicted from which a magnification of the best 50 multi-starts is indicated by the blue box. Red dots denote the starts converged to the global optimum within a small numerical margin. B: Scatter plot for the overall agreement of experimental data and model fit. C-D: Comparison of selected experimental data and model fits. Time and dose response data obtained using immunoblotting indicate the mean and standard deviation of three biological experiments. For visualization in C and D, experimental measurements were scaled to model simulation using the estimated scaling factors to overlay the response to different experimental conditions. Additional data and model fits are provided in S3 Fig and S1 Text, Section 2.

**Fig 3:**
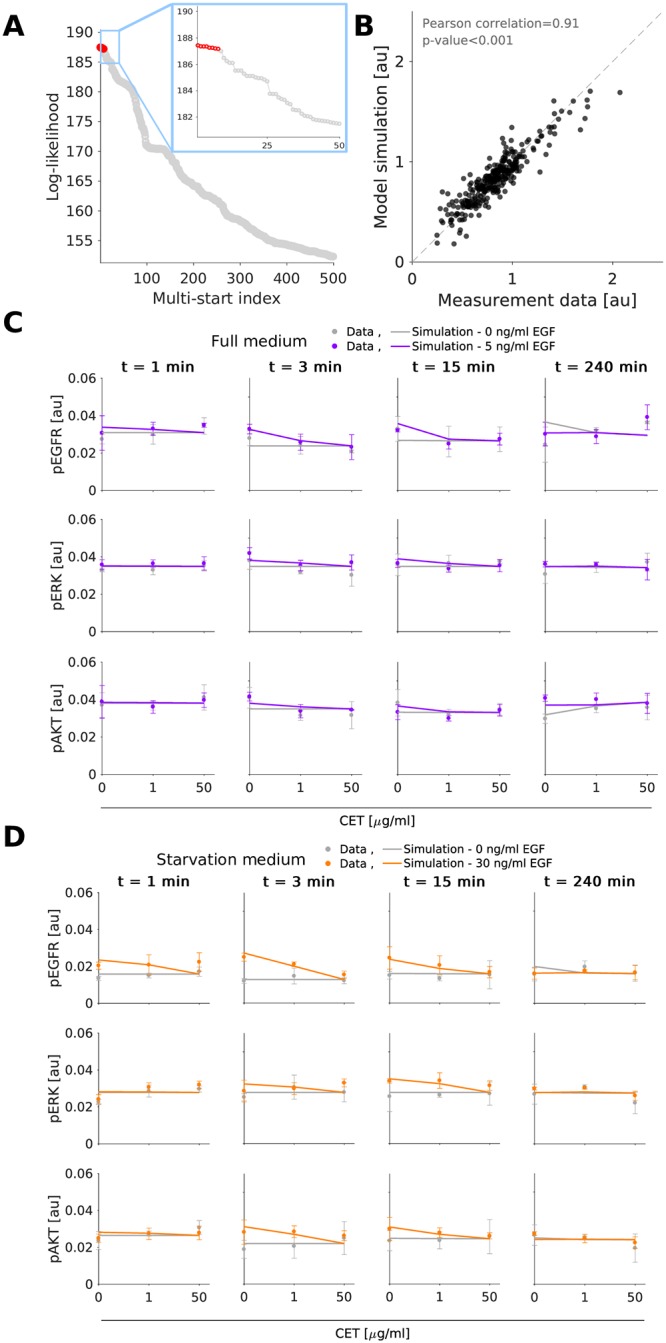
Experimental data and model fit for the gastric cancer cell line Hs746T. A: Waterfall plots for multi-start local optimization. The best 500 out of 2000 runs are depicted from which a magnification of the best 50 multi-starts is indicated by the blue box. Red dots denote the starts converged to the global optimum within a small numerical margin. B: Scatter plot for the overall agreement of experimental data and model fits. C-D: Comparison of selected experimental data and model fit. Time and dose response data obtained using immunoblotting indicate the mean and standard deviation of three biological experiments. For visualization in C and D, experimental measurements were scaled to model simulation using the estimated scaling factors to overlay the response to different experimental conditions. Additional data and model fits are provided in S4 Fig and S1 Text, Section 2.

We determined the maximum likelihood estimates of the model parameters, e.g. rate constants, using multi-start local optimization [31, 32] (see Materials and methods). The estimation was performed individually for MKN1 and Hs746T cells. For MKN1 cells, the optimizer converged reliably. This is reflected in the clear plateau in the waterfall plot (Fig 2A). For the optimal parameters we observed a good agreement of MKN1 data and model fit (Fig 2B–2D) (Pearson correlation *ρ* = 0.95). For Hs746T cells, the results of the multi-start optimization do not show clear plateaus, suggesting that the objective function possesses a large number of local optima or flat regions (Fig 3A). But even so, the best fit found provides an accurate description of the data (Fig 3B–3D) (Pearson correlation *ρ* = 0.91) and the best 100 fits achieve similar correlations (S7 Fig). Furthermore, the best 10 fits are statistically not distinguishable from the best fit [33]. A potential source of the convergence problems for Hs746T cells is the low signal-to-noise ratio, which is caused by the limited response to EGF and cetuximab treatment (see, e.g., Fig 3C and 3D) since this is a non-responder cell line. Yet, despite the low signal-to-noise ratio several changes are statistical significant (see analysis by [9] for a part of the published data). As the model fits provided a good description of most data points, the parameter estimation confirmed that the proposed pathway model is a good candidate for further analysis.

### Model-based cell line comparison predicts causal differences beyond mutations and expression patterns

The available experimental data (Figs 2 and 3, S3 and S4 Figs, and S1 Text, Section 2) show a pronounced difference in the response of the cell lines to EGF and cetuximab treatment. Potential sources of this behavior are differences in mutation patterns, protein expression levels/abundances and reaction fluxes (due to an influence of latent components between the cell lines). While we did not perform a comprehensive quantification of the protein abundances, selected measurements and transcriptome data indicate substantial differences between the cell lines.

To identify important differences in the reaction fluxes, we compared the parameter estimates obtained for the individual cell lines. These parameter estimates should reflect changes on the biochemical level between the cell lines. In the comparison, we only consider parameters associated with single model reactions summarizing multi-step signaling processes.

Examples include the indirect activation of ERK by RAS, which is described by one reaction in the considered pathway model. As the expression levels of the proteins involved in the intermediate reactions—in this case RAF and MEK (Fig 1A)—can vary, the reaction rate constant linked to each multi-step process can easily differ between cell lines. In contrast, for direct reactions, such as the binding of EGF to EGFR, the reaction rate constant should be identical for both cell lines. For details on the classification for the reactions we refer to the S1 Text, Section 3.

**Fig 4:**
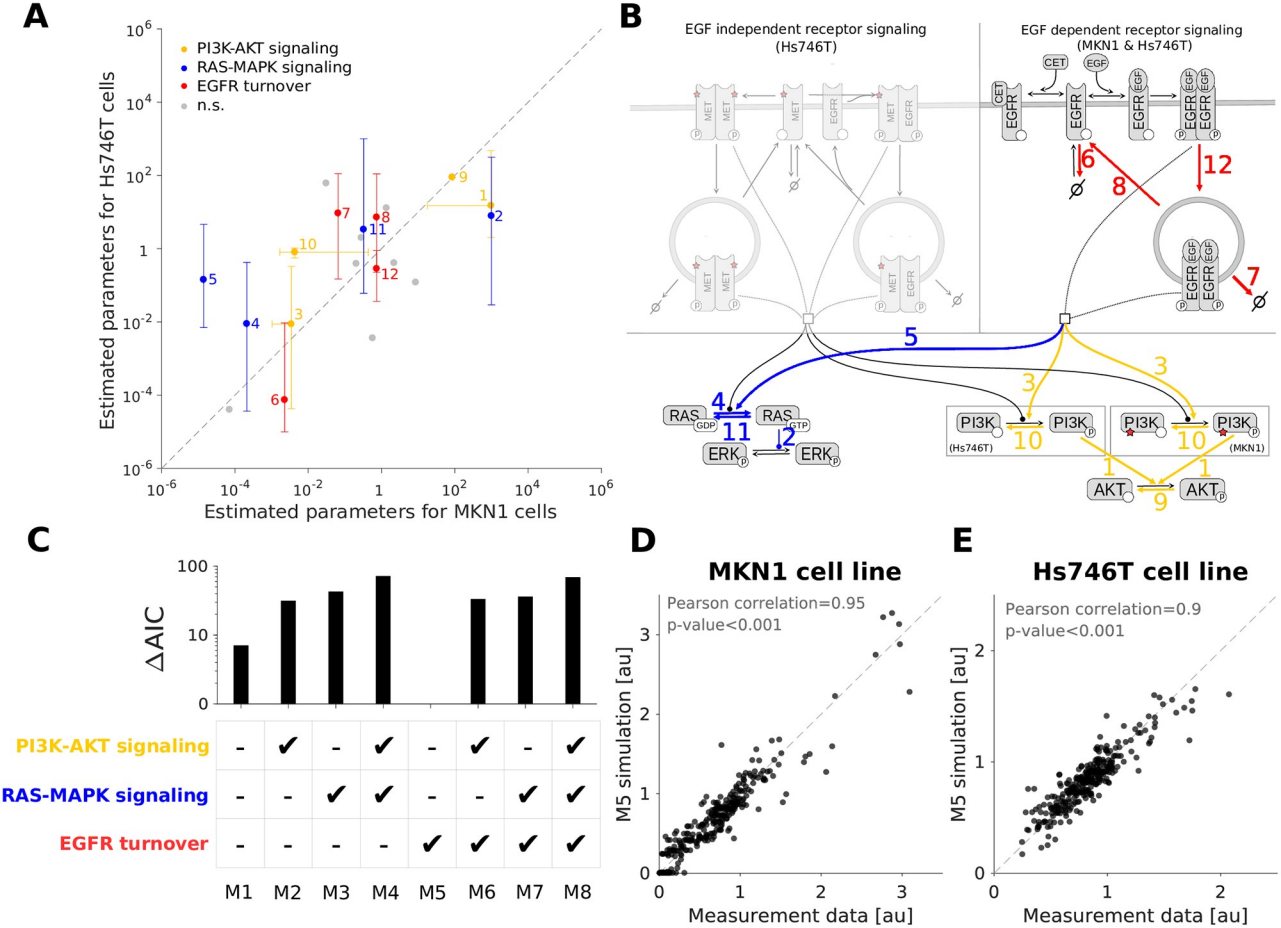
Identification of cell line specific parameters. A: RAS-MAPK and PI3K-AKT signaling pathways as well as EGFR turnover dynamics as possible cell line specificity candidates. Dots and bars depict the median and one standard deviation (68% percentil interval) of the top 100 log10-transformed parameter estimates obtained by the optimization. Values obtained from MKN1 and Hs746T cells are shown along the X and Y axis, respectively. Coloring indicates significantly different parameter pairs (*p* < 0.05). Gray points indicate non-significantly different and direct reactions (n.s.). B: Highlighted cell line specificity candidates in the model overview. Coloring and numeric labeling correspond to Fig 4A. C: Model selection using AIC shows that only changing the receptor turnover dynamics results in the best model. D: Scatter plot for the overall agreement of experimental data and combined model fit for the MKN1 cell line. Results correspond to the best model (M5). E: Scatter plot for the overall agreement of experimental data and combined model fit for the Hs746T cell line. Results correspond to the best model (M5). The fits for the selected model (M5) are provided in S1, S5 and S6 Figs, and S1 Text, Section 2.

For the comparison we considered the best 100 parameter vectors obtained by multi-start local optimization for MKN1 and Hs746T cell lines. Statistical testing for cell line specificity of parameters was performed using one-way-ANOVA suggesting significant (*p* < 0.05) differences for 12 out of 20 estimated kinetic rates (Fig 4A). The mapping of the findings on the pathway visualization revealed potential differences in (i) RAS-MAPK signaling, (ii) PI3K-AKT signaling, and (iii) EGFR turnover including internalization, degradation and recycling (Fig 4B, S1 Table and S1 Text, Section 4). In addition, mutated MET (MMET) and mutated PI3K can cause differences between cell lines as they are each only present in one of the cell lines.

The comparison of the optimal parameter values found for the different cell lines provides a conservative evaluation. Indeed, not all differences between cell lines might be required to fit the data (see [34]). To assess the relevance of differences in modules (i)-(iii), we fitted the dataset for the MKN1 and Hs746T cell lines simultaneously. Therefore, a collection of mathematical models was developed accounting for cell line specific mutations and protein abundances, as well as all possible combinations of differences of parameters belonging to modules (i), (ii) and (iii). All remaining kinetic rates were assumed to be identical for the cell lines. In total, eight different models were constructed accounting for all possible combinations. For each candidate model, a multi-start local optimization run was performed using the complete dataset. The combination of molecular data for responder and non-responder cell lines provides addition constraints for the parameters and increases the number of data points per estimated parameter.

Model selection using AIC, indicated that the model including cell line specific differences only in the EGFR turnover dynamics provides the best qualitative description of the experimental data (Fig 4C). This agrees with previous findings reporting the presence of FBXW7 p.R465C mutation in MKN1 cells, but not in Hs746T cells. FBXW7 ubitiquinates EGFR, leading to changes in the turnover/degradation of EGFR between the cell lines [35]. The resulting model provides an accurate description of the experimental data for the responder (Fig 4D) and the non-responder (Fig 4E) cell lines. We evaluated if the fitting can be improved by including additional processes such as the negative feedback regulation of RAS activity by phosphorylated ERK [36]. However, the AIC value did not improve (see S8 Fig and S1 Text, Section 7). The subsequent analyzes are based on this model.

### Integrated modeling of responder and non-responder cell lines yields reliable predictions

The selected pathway model describing the experimental data for the cell lines MKN1 and Hs746T possesses 230 parameters in total. 57 parameters are associated with the reaction kinetics and protein abundances (for wild-type and mutant proteins), and 173 are parameters related to the observations (i.e. scaling constants). To assess the identifiability of the kinetic parameters as well as differences between cell lines and media, we computed the profile likelihoods. We did not calculate the profiles for the observation parameters as they differ between single experiments and are not relevant for the model dynamical response.

**Fig 5:**
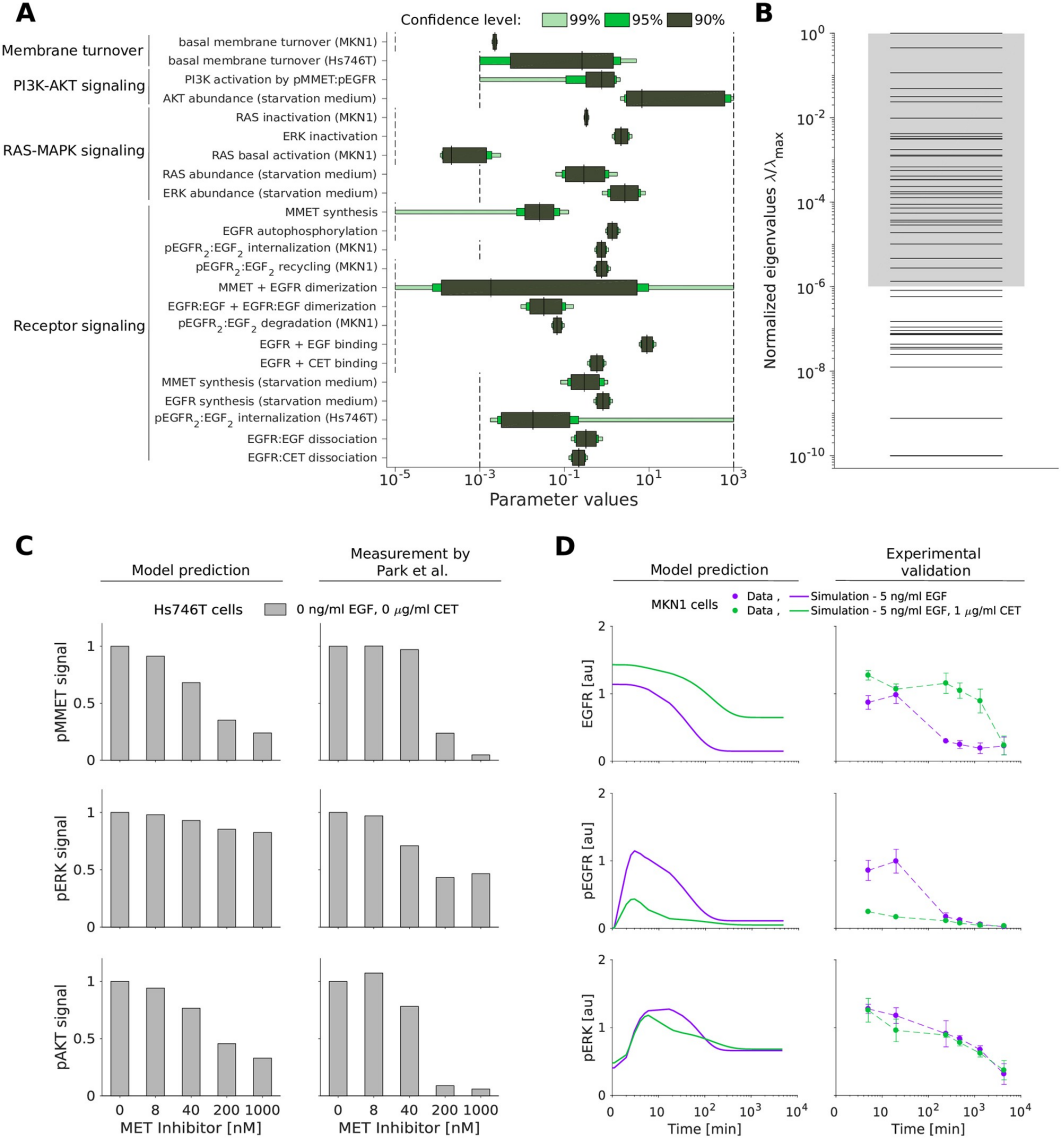
Uncertainty of the parameter estimates does not impair accurate model predictions. A: Confidence intervals for the identifiable parameters. The confidence intervals corresponding to the confidence levels 90%, 95% and 99% are shown. Parameter bounds used for optimization are indicated in black dashed lines. B: Eigenvalue spectrum of the FIM for the dynamical parameters. The spectra have each been normalized by their largest eigenvalue (λ_max_). Gray shading indicates a spreading of less than six orders of magnitude. C: *In silico* prediction and experimental validation of MET inhibitor response in Hs746T cells. Validation data for the MET inhibitor KRC-00715 extracted from [38, Fig 2]. The signal was normalized with respect to the untreated condition. D: Model prediction (left) and experimental validation (right) of long-term response to EGF and the combination of EGF with cetuximab in MKN1 cells. Western blot images corresponding to D are provided in S10 Fig.

Profile likelihoods provide a maximum projection of the likelihood on the individual parameters (Materials and methods). The analysis of the profile likelihoods revealed that 23 out of 57 parameters are practically identifiable, meaning that the 90% confidence intervals are finite (Fig 5A). Most of the identifiable parameters take part in the EGFR dynamics module, involving processes such as internalization, degradation, ligand binding and dimerization. The remaining parameters are practically non-identifiable, meaning that lower and/or upper bounds could not be found for the defined confidence level. In particular, parameters related to MET signaling are practically non-identifiable. This is not unexpected as MMET is not directly observed.

We complemented the profile likelihood calculation with an evaluation of the Fisher Information Matrix (FIM) at the maximum likelihood estimate. The eigenvalue spectrum of the FIM for the kinetic parameters and initial conditions spans many order of magnitude (Fig 5B) implying sloppiness [37].

Interestingly, there are 50 eigenvalues which differ substantially from numerical zero, meaning that the number of constraint directions in parameter space—given by the eigenvectors—is larger than the number of identifiable parameters. This can happen if individual parameters are non-identifiable but functions of several parameters (e.g. sums, differences or ratios) are identifiable. As the detailed analysis of this using profile likelihoods is computationally demanding, the analysis of the FIM provides a first glimpse.

While the estimates of many parameters appear reliable, the insights which can be obtained by studying the parameter values is limited. As many parameters in the model represent multi-step processes, there is no clear biological counterpart. This is different for the state variables and outputs of the model, allowing for model-based predictions. To assess the predictive power of the model despite the sloppy eigenvalue spectrum and the non-identifiable parameters, we simulated the system in additional experimental conditions, i.e. in conditions not used for the fitting. Firstly, we evaluated whether the model can despite the non-identifiabilities in the MET signaling dynamics predict published experimental data for the response of Hs746T cells to selected MET inhibitors. Therefore, the inhibitor was implemented in the model, and simulation was performed using published parameters (for details on the model implementation refer to S1 Text, Section 3). We found that the model qualitatively predicts the reduction of pMMET and pAKT levels observed by [38] (Fig 5C). Secondly, we predicted the state of MKN1 cells beyond the first 240 min for which experimental data are available. We found that the model provides reasonable predictions for long-time response of EGFR, pEGFR and pERK (Fig 5D). The quality of these predictions profits from the model topology as well as the estimated parameter values (see S9 Fig and S1 Text, Section 8). Accordingly, our analysis showed that the model structure and parameter estimates are reasonable and that the model is able to predict the considered validation experiments.

### Mathematical model reliably predicts response and resistance factors

**Fig 6:**
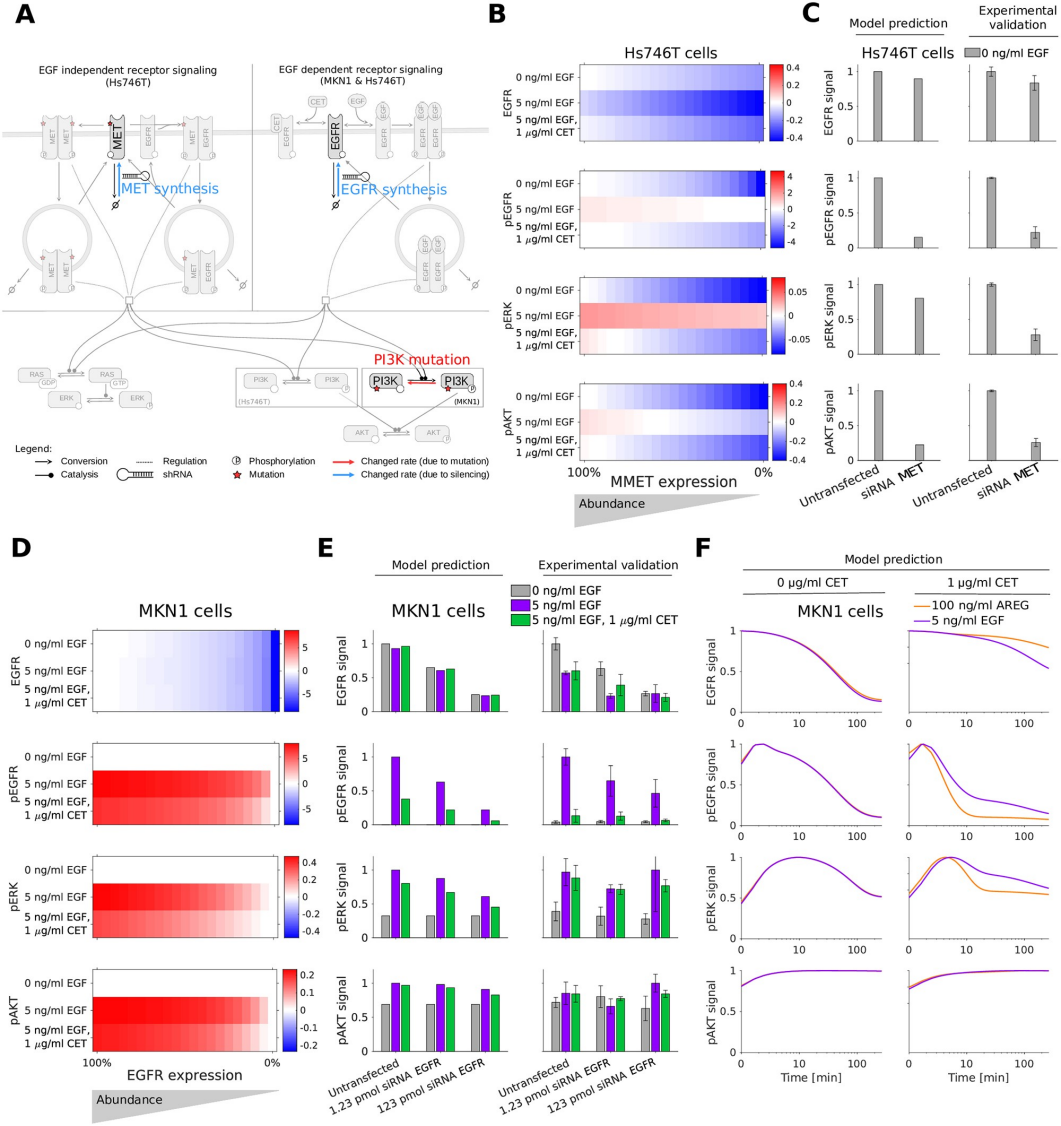
Model prediction of response and resistance factors. A: Model overview showing changed rates for EGFR and MMET synthesis, and PI3K mutation. B: *In silico* screening for MMET expression silencing in Hs746T cell line for unstimulated, EGF, and EGF in combination with cetuximab treatment at 3 min. C: Model prediction and experimental validation of MMET expression silencing using siRNA MET at 3 min. For the simulation, the MMET synthesis rate was scaled with the measured MMET reduction achieved by siRNA treatment in the untreated (no stimulation) condition. D: *In silico* screening for EGFR expression silencing in MKN1 cell line for unstimulated, EGF, and EGF in combination with cetuximab treatment at 5 min. For the simulation, the EGFR synthesis rate was scaled with the measured EGFR reduction achieved by siRNA treatment in the untreated (no stimulation) condition. E: Model prediction and experimental validation of EGFR expression silencing using siRNA EGFR at 5 min. F: Model prediction of time response of AREG stimulation (black line) shows higher sensitivity to cetuximab treatment compared to EGF (red line) in MKN1 cells. B-F: The signal is normalized with respect to the maximum activity level for each observed component. Western blot images corresponding to C and E are provided in S11 and S12 Figs.

The developed mathematical model provides a screening tool for the rapid assessment of potential response and resistance factors. Here, we demonstrate this by studying the validity of the predictions for several established factors (Fig 6A).

The evident differences between the responder cell line, MKN1, and the non-responder cell line, Hs746T, are the mutation patterns. The experimental data for MKN1 cells showed that PIK3CA p.E545K is not a resistance factor. However, our model predicts the association of PIK3CA overexpression (due to p.E545K mutation) with an insensitivity of pAKT to cetuximab treatment (S2 Fig). This is difficult to test experimentally, but consistent with the finding that PIK3CA mutation is a source of acquired cetuximab resistance in metastatic colorectal cancer patients [39]. The model predictions suggest the same for gastric cancer, although “PIK3CA mutations were not significantly associated with any clinical, epidemiologic, or pathologic characteristic” in gastric cancer patients obtaining non-targeted therapy [40].

The MET exon 14 juxtamembrane splice site mutation found in Hs746T cells inhibits the degradation of MET receptor, prolonging its oncogenic activity [41]. MET activation is an established resistance factor for cetuximab treatment in gastric cancer [24]. Indeed, our model predicts that a knockdown of mutant MET reduces Hs746T cell proliferation and survival signaling via ERK and AKT (Fig 6B). To validate the prediction, MET was silenced and quantitative immunoblotting was performed. We found that the model provides accurate quantitative predictions for EGFR, pEGFR, pERK and pAKT (Fig 6C and S9 Fig) (Pearson correlation *ρ* = 0.872) although the down-regulation of pERK is slightly underestimated. For detailed information about the modeling of MET silencing refer to S1 Text, Section 3.

Beyond mutations, amplifications and expression changes have been reported as response and resistance factors. In particular the abundance of EGFR has been reported to be associated with cetuximab response in gastric cancer [24, 42]. Our model predicts that reducing EGFR expression levels in MKN1—a cell line overexpressing EGFR—decreases the level of pEGFR, pERK and pAKT (Fig 6D). Interestingly, the effect on downstream signaling was predicted to be relatively small. We tested this prediction by silencing EGFR expression and found a good agreement with experimental data (Fig 6E and S9 Fig) (Pearson correlation *ρ* = 0.915). The result implies that the dependence of ERK and AKT activity on EGFR activity is limited. For detailed information about the modeling of EGFR silencing refer to S1 Text, Section 3.

Beyond the expression of EGFR, the abundance of the EGFR ligand AREG has been shown to correlate positively with cetuximab response [24]. As the biochemical processes underlying AREG- and EGF-induced activation of EGFR signaling are similar [43], we employed the same model for AREG and EGF treatment. Following the literature [44], we assumed that AREG has an EGFR affinity about 50 times lower than EGF. We neglected that AREG stimulation leads to EGFR recycling while EGF promotes EGFR degradation [45]. The resulting model predicted in the absence of MET activation, i.e. in MKN1 cells, that for higher AREG levels cetuximab achieves a higher reduction in EGFR and ERK phosphorylation (Fig 6F). As a consequence of PI3K mutation in MKN1 cells, AKT activation is insensitive to changes in the receptor signal. This model prediction is in line with the published experimental data in [24].

## Conclusion

Mechanistic ODE models are widely used for the integration of experimental data and the analysis of causal relations. Furthermore, recent studies demonstrate their potential for the identification of biomarkers [6, 14, 15]. To render this approach available for gastric cancer, we developed a mechanistic model of signaling in gastric cancer. The model describes the dynamics of the EGFR, ERK and AKT signaling pathways in response to EGF and cetuximab treatment, accounting for mutation patterns and protein expression levels. The proposed model provides a more detailed description than the available logical model [22] by capturing individual biochemical reactions and encoding the wild-type and mutated genes. To the best of our knowledge, the proposed model is the first mechanistic model tailored to gastric cancer signaling.

To assess the predictive power of the model, we performed a broad spectrum of analyzes. First, we used the model to study causal differences between the cetuximab responder cell line, MKN1, and non-responder cell line, Hs746T. The analysis suggested the presence of a MET mutation as well as differences in receptor internalization and recycling as key factors for the response to cetuximab treatment. Second, we validated the predicted response to a MET inhibitor (KRC-00715) as well as to long-term EGF and EGF in combination with cetuximab treatment. Third, we employed the model to predict response and resistance factors. The predicted role of the EGFR abundance as well as MMET were experimentally confirmed by knockdown experiments. The array of successful validations suggests that the model has the potential to be used as a kickoff tool for guiding biomarker discovery.

While all tests were positive, the parameters and predictions of the proposed models are subject to uncertainties. Less than 50% of the parameters were practically identifiable, implying that addition of experimental data is required. In particular, measurements of MMET activity would be beneficial, as well as the absolute quantification of expression and phosphorylation levels. Furthermore, a detailed experimental analysis of the impact of gene expression on cetuximab response using isogenic cell lines would provide valuable information about signal processing. Complementary, the model provides only a crude description of the core pathways and extensions might become necessary. Specifically, the adaptor proteins, intermediate kinases and the effect of multi-site phosphorylation should be included. Furthermore, additional members of the HER family and additional receptor tyrosine kinases, such as AXL, could be included. A template for the refinement could be provided by the Atlas of Cancer signaling Networks [46] or the large-scale mechanistic model we recently introduced [15]. However, all model extensions should be counterbalance with acquisition of additional experimental data for gastric cancer cell lines.

For model-based patient stratification, the model also needs to be extended to account for antibody-dependent cellular cytotoxicity (ADCC). ADCC is—besides the binding of cetuximab to EGFR and the inhibition of receptor dimerization and intracellular signal transduction—the second mode of active of cetuximab. ADCC provokes immune cell-mediated killing of tumor cells [8]. Since the ADCC effect cannot be observed in vitro, patient material would be required to study this effect.

In conclusion, the proposed model provides a potentially valuable tool for gastric cancer research in the context of cetuximab treatment. It aggregates the information of a comprehensive list of publications and describes a large set of data points. In the future, it might be used to integrate additional data, e.g. from different cell lines and drugs.

## Materials and methods

### Cell lines and cell culture conditions

For the establishment of the model, the human gastric cancer cell lines Hs746T and MKN1 were used and cultured as reported by [9].

### Western blot analyzes

Western blot analyzes to set up the model were performed as reported earlier [9, 24, 35]. The cells were treated for indicated times with 0.05, 0.1, 1, 10 or 50 *μ*g/ml cetuximab and/or 5, 30 or 100 ng/ml EGF.

The following antibodies were used: anti-EGFR (Cell signaling Technology (CST), distributed by New England Biolabs in Frankfurt, Germany; #2232; dilution 1:1,000 in 5% BSA TBS-T), anti-pEGFR Y1068 (Life Technologies, Darmstadt, Germany; #44788G; dilution 1:2,000 in 5% milk TBS-T), anti-MET (Cell signaling Technology (CST), distributed by New England Biolabs in Frankfurt, Germany; #8198; dilution 1:1,000 in 5% BSA TBS-T), anti-*α*-tubulin (Sigma-Aldrich, Steinheim, Germany; #T9026; dilution 1:10,000 in 5% milk TBS-T), anti-*β*-actin (Sigma-Aldrich; A1978; dilution 1:5,000 in 5% milk TBS-T), anti-mouse IgG (GE Healthcare, distributed by VWR in Ismaning, Germany; #NA931; dilution 1:10,000 in 5% milk TBS-T) and anti-rabbit IgG (CST; #7074; dilution 1:2,000 in TBS-T).

For quantification, the signals were densitometrically analyzed using ImageJ 1.44p Software [47].

### Knockdown experiments

To generate a transient knockdown of EGFR in the cell line MKN1 and a knockdown of MET in Hs746T cells, siRNA was used. Cells were cultured overnight in rich medium, before the medium was exchanged for antibiotic-free medium. A specific FlexiTube GeneSolution (Qiagen, GS1956 for EGFR, GS4233 for MET) was used, containing four gene specific, preselected siRNAs. The AllStars Negative Control siRNA and AllStars Negative Control siRNA AF488 were used as negative controls. Cells were transfected with Lipofectamine 2000 according to the manufacturer’s instruction. After a transfection time of 4 h (EGFR knockdown in MKN1 cells) or 24 h (MET knockdown in the cell line Hs746T), the medium was replaced with rich medium. The transfection was checked on protein level by western blot analyzes. Therefore, samples were collected 24 hours after the transfection. For analysis of EGFR, MAPK and AKT activation after EGFR knockdown, cells were treated for 5 minutes with 5 ng/ml EGF or the combination of 1 *μ*g/ml cetuximab plus 5 ng/ml EGF.

### Mechanistic modeling

The dynamics of the biochemical reaction networks were modelled using systems of ODEs,

$$\begin{array}{c} \frac{dx}{dt}=Sv\left(x,\theta \right),\;x\left(0\right)=x_{0}\left(\theta \right) \end{array}$$

in which *x* denotes the concentration vector of the biochemical species, *S* denotes the stoichiometric matrix, *v*(*x*, *θ*) denotes the reaction flux vector and *θ* denotes the parameter vector. The parameters are, e.g., rate constants of synthesis or degradation reactions. As the cells start from an unperturbed state, the initial condition *x*_0_(*θ*) is the unperturbed steady state of the ODE.

To infer causal differences between responder and non-responder cell lines, we allowed for differences between the parameters of the cell lines. Following the work of [34], we modelled the difference by the additional parameters *ξ*_*i*_,

$$\begin{array}{c} \theta_{i,\text{Hs}746\text{T}}=\exp\left(\xi_{i}\right)\theta_{i,\text{MKN1}}. \end{array}$$

The parameters *ξ*_*i*_ denotes the log-fold change for the *i*-th parameter. If *ξ*_*i*_ is zero, the parameters of the two cell lines are identical. We fixed *ξ*_*i*_ to zero for all parameters which are associate with direct biochemical interactions, e.g. binding rates, which should be conserved between cell lines. Only for expression levels and indirect interactions, i.e. simplification of multi-step processes, we allowed for *ξ*_*i*_ ≠ 0 and therefore estimated along with *θ* → (*θ*, *ξ*). Similar to the modeling of differences between cell lines, we modelled differences between cell culture media (rich and starvation medium). Here, only differences in the expression levels are allowed. Specific information about parameters are provided in the S1 Text, Section 5, and S2 Table.

The concentration of biochemical species were linked to the observables *y*(*t*) by observation functions *h*_*j*_,

$$\begin{array}{c} {\bar{y}}_{jk}=h_{j}\left(x\left(t_{k}\right),\theta \right)+\epsilon_{j}, \end{array}$$

in which *j* indexes the observable and *k* indexes the time point. We assumed independent and additive normally distributed measurement noise ${∊}_{j}∼N\left(0,\sigma_{j}^{2}\right)$. To adapt the scale of different replicates and standard deviation of the experimental data, we employed mixture modeling. The standard deviations were allowed to differ between observables but were assumed to be independent of time and treatment condition for each individual experimental setup (e.g. a set of Western blots). We fixed *σ*^2^ to the estimated standard deviations and therefore reduced the complexity of the optimization problem [54]. Details are provided in the S1 Text, Section 1.

### Parameter estimation

We performed maximum likelihood estimation by minimizing the negative log-likelihood function,

$$\begin{array}{c} \theta^{*}=\text{argmin}\{-\log\left(L\right)≔\frac{1}{2}\sum_{i}{\left(\frac{y_{i}-h\left(x\left(t_{j}\right)\right)}{\sigma_{i}}\right)}^{2}+\text{const}.\}, \end{array}$$ (1)

in which *x*(*t*_*j*_) denotes the solution of the ODE model for the parameter vector *θ*.

Numerical optimization was conducted using multi-start local optimization. This approach has been shown to outperform most global optimization methods and achieves a performance comparable with sophisticated hybrid optimization methods [31, 48]. For each fitting problem, initial parameters were generate using Latin hypercube sampling. The local optimization was performed by trust-region based algorithms implemented in the MATLAB function *lsqnonlin*. For the optimization, parameters were log_10_-transformed to improve numerical properties, e.g. improvement of convexity [49] and computational efficiency [48, 50]. For detailed information about the optimization options refer to S1 Text, Section 6.

For the fitting of the individual cell lines, we generated 1000 starting points for local optimization. For the model selection, 100 starting points were used instead. The model selection was based on the AIC values for each model alternative. Following the work of [51], a difference of 10 was considered to be substantial.

For the assessment of differences between cell lines, we employed an iterative process instead of the regularization approach proposed by [34]. We also tested the latter, but the optimization did not converge and the penalization did not achieve a clear model reduction.

### Uncertainty analysis

The uncertainty of the model parameters was assessed by analyzing the eigenvalue spectrum of the FIM at the maximum likelihood estimate. The parameter estimation results were considered sloppy if minimum and maximum eigenvalue differed by more than six orders of magnitude [37].

The local analysis provided by the FIM was complemented by computing the profile likelihoods [52]. From the profile likelihoods, we computed the confidence intervals for each individual parameter disregarding scaling constants. Parameters with unbounded confidence intervals for a significance level of 0.1 were considered as practically non-identifiable.

### Software

The model formulation, parameter estimation and uncertainty analysis was performed in the MATLAB toolbox Data2Dynamics (https://github.com/Data2Dynamics/d2d, commit 9b1c3556) [32]. The parameter confidence intervals and the visualization of parameter uncertainties was carried out using the MATLAB toolbox PESTO (https://github.com/ICB-DCM/PESTO, commit 8278a1a) [53]. For image quantification, we used ImageJ software [47].

## Supporting information

S1 Fig Experimental data and combined model fit for the best model M5. A-B: Comparison of selected experimental data and model fit for Hs746T cell line. C-D: Comparison of selected experimental data and model fit for MKN1 cell line. A-D: Time and dose response data obtained using immunoblotting indicate the mean and standard deviation of three biological experiments. Experimental measurements were scaled to model simulation using the estimated scaling factors. E: Waterfall plots for multi-start local optimization. The best 200 out of 1000 runs are depicted from which a magnification of the best 50 multi-starts is indicated by the blue box. Red dots denote the starts converged to the global optimum within a small numerical margin. Additional data and model fits are provided in S5 and S6 Figs.
(TIF)

S2 Fig Expression of wild-type PI3K recovers pAKT levels sensitivity to cetuximab treatment in MKN1 cell line. Model prediction of time response of wild-type PI3K (black line) shows a reduction in AKT activity compared to PI3K p.E545K (red line), which remains insensitive. The signal is normalized with respect to the maximum activity level for each observed component.
(TIF)

S3 Fig Model-data comparison for the MKN1 cell line, for datasets not depicted in main manuscript Fig 2. A-B: Time response to different EGF concentrations in starvation culture media (HM). C: Dose response to EGF and cetuximab stimulation at 3 min in rich culture media (FM). D: Dose response to EGF and cetuximab stimulation at 3 min in starvation culture media (HM). E: Dose response to EGF and cetuximab stimulation at 0, 1, 15 and 30 min in full (FM) and starvation culture media (HM). C-E: Specific EGF and cetuximab concentrations are shown along the X axis.
(TIF)

S4 Fig Model-data comparison for the Hs746T cell line, for datasets not depicted in main manuscript Fig 3. A: Time response to EGF stimulation in starvation culture media (HM). B: Time response to EGF stimulation in full (FM) and starvation culture media (HM). C: Time response to EGF and cetuximab stimulation in rich culture media (FM). D: Dose response to EGF and cetuximab stimulation at 3 min in rich culture media (FM). E: Dose response to EGF and cetuximab stimulation at 3 min in starvation culture media (HM). D-E: Specific EGF and cetuximab concentrations are shown along the X axis.
(TIF)

S5 Fig Model-data comparison for the combined fitting of MKN1 and Hs746T cell lines, for datasets not depicted in main manuscript Fig 4 and S1 Fig. Model fits for the best model (M5). A: Time response to EGF stimulation in starvation culture media (HM). B: Dose response to EGF and cetuximab stimulation at 3 min in rich culture media (FM). C: Dose response to EGF and cetuximab stimulation at 3 min in starvation culture media (HM). D: Time response to EGF stimulation of Hs746T cells in full (FM) and starvation culture media (HM). A-C: Experimental data for both cell lines. B-C: Specific EGF and cetuximab concentrations are shown along the X axis.
(TIF)

S6 Fig Model-data comparison for the combined fitting of MKN1 and Hs746T cell lines, for datasets not depicted in main manuscript Fig 4 and S1 Fig. Model fits for the best model (M5). A: Time response to EGF and cetuximab stimulation of MKN1 cells in starvation culture media (HM). B: Time response to EGF and cetuximab stimulation of Hs746T cells in rich culture media (FM). C: Dose response to EGF and cetuximab stimulation at 0, 1, 15 and 30 min of MKN1 cells in rich (FM) and starvation culture media (HM). Specific EGF and cetuximab concentrations, time points and culture media, are shown along the X axis.
(TIF)

S7 Fig Overview on model and data correlation for multiple parameter sets on the individual cell line models. Boxplots for the overall agreement of experimental data and model fits for, A: the best 10 parameter sets, B: the best 50 parameter sets, and C: the best 100 parameter sets. The individual model fits for Hs746T and MKN1 cells are shown.
(TIF)

S8 Fig Comparison of model with and without feedback. A: Schematic of model including negative feedback regulation from ERK to RAS. B: Differences of AIC values for the model and the best AIC. The parameter estimation results for both models were obtained using 300 local optimization runs. The analysis suggested that the model without feedback is more consistent with the experimental data. C: Waterfall plot for multi-start local optimization. The best 50 out of 300 runs are depicted. Red dots denote the starts converged to the global optimum within a small numerical margin. D: Correlation of simulation results for model M5 (optimal model without feedback) and the model with feedback.
(TIF)

S9 Fig Validation of the parameter sets. A: Scatter plot of the likelihood ratios for the initial parameter guesses, *θ*_*initial*_, and their corresponding final optimized values, *θ*_*optimal*_. The likelihood ratio was calculated with respect to the best/maximum likelihood estimate found during the multi-start local optimization. The 20 best parameter sets are shown paired with their initial guesses. B-D: Boxplots of the agreement between the validation data and model simulation for the initial parameter guesses, *θ*_*initial*_, and their corresponding final optimized values, *θ*_*optimal*_. The agreement is shown in terms of Spearman’s correlation coefficients. The 20 best parameter sets are shown paired with their initial guesses. The validation datasets shown are (B) MET inhibition, (C) long-term kinetic response in MKN1 cells, and (D) silencing of EGFR and MET expression.
(TIF)

S10 Fig Western blots for long-term response to EGF and the combination with cetuximab treatment in MKN1 cells. Related to Fig 5D in the main manuscript.
(TIF)

S11 Fig Western blots for MET expression silencing. Related to Fig 6C in the main manuscript.
(TIF)

S12 Fig Western blots for EGFR expression silencing. Related to Fig 6E in the main manuscript.
(TIF)

S1 Text Supporting information for the optimization and model reactions. (PDF)

S1 Table Reaction rate candidates for cell-line specificity. The following kinetic rates were identified as significantly different between the cell lines by the ANOVA statistical test. All kinetic rates belonging to the same module were simultaneously tested (not individually) resulting in 8 different model candidates.
(PDF)

S2 Table Literature values used as prior information in the parameter estimation. Prior mean values were converted to 10-logarithmic scale and used for the parameterization of the model. Note that the same kinetic rate can appear in different reactions, e.g., receptor endocytosis in R10, R22 and R34.
(PDF)

## References

[1] Bray F, Ferlay J, Soerjomataram I, Siegel R, Torre L, A J. Global cancer statistics 2018: GLOBOCAN estimates of incidence and mortality worldwide for 36 cancers in 185 countries. CA Cancer J Clin. 2018;. 10.3322/caac.21492 30207593

[2] Lordick F, Allum W, Carneiro F, Mitry E, Tabernero J, Tan P, et al Unmet needs and challenges in gastric cancer: the way forward. Cancer Treat Rev. 2014;40(6):692–700. 10.1016/j.ctrv.2014.03.002 24656602

[3] Bang YJ, Van Cutsem E, Feyereislova A, Chung HC, Shen L, Sawaki A, et al Trastuzumab in combination with chemotherapy versus chemotherapy alone for treatment of HER2-positive advanced gastric or gastro-oesophageal junction cancer (ToGA): a phase 3, open-label, randomised controlled trial. Lancet. 2010;376(9742):687–97. 10.1016/S0140-6736(10)61121-X 20728210

[4] Lordick F, Kang YK, Chung HC, Salman P, Oh SC, Bodoky G, et al Capecitabine and cisplatin with or without cetuximab for patients with previously untreated advanced gastric cancer (EXPAND): a randomised, open-label phase 3 trial. Lancet Oncol. 2013;14(6):490–9. 10.1016/S1470-2045(13)70102-5 23594786

[5] Li S, Schmitz KR, Jeffrey PD, Wiltzius JJW, Kussie P, Ferguson KM. Structural basis for inhibition of the epidermal growth factor receptor by cetuximab. Cancer Cell. 2005;7(4):301–11. 10.1016/j.ccr.2005.03.003 15837620

[6] Hass H, Masson K, Wohlgemuth S, Paragas V, Allen JE, Sevecka M, et al Predicting ligand-dependent tumors from multi-dimensional signaling features. npj Syst Biol Appl. 2017;3(1):27 10.1038/s41540-017-0030-3 28944080 PMC5607260

[7] Singh B, Carpenter G, Coffey RJ. EGF receptor ligands: recent advances. F1000 Research. 2016;. 10.12688/f1000research.9025.1 PMC5017282 27635238

[8] Hara M, Nakanishi H, Tsujimura K, Matsui M, Yatabe Y, Manabe T, et al Interleukin-2 potentiation of cetuximab antitumor activity for epidermal growth factor receptor-overexpressing gastric cancer xenografts through antibody-dependent cellular cytotoxicity. Cancer Science. 2008;. 10.1111/j.1349-7006.2008.00821.x PMC11159884 18422755

[9] Keller S, Kneissl J, Grabher-Meier V, Heindl S, Hasenauer J, Maier D, et al Evaluation of epidermal growth factor receptor signaling effects in gastric cancer cell lines by detailed motility-focused phenotypic characterization linked with molecular analysis. BMC Cancer. 2017;17(1):845 10.1186/s12885-017-3822-3 29237412 PMC5729506

[10] Poste G. Bring on the biomarkers. Nature. 2011;. 10.1038/469156a 21228852

[11] Barretina J, Caponigro G, Stransky N, Venkatesan K, Margolin AA, Kim S, et al The Cancer Cell Line Encyclopedia enables predictive modelling of anticancer drug sensitivity. Nature. 2012;483(7391):603–607. 10.1038/nature11003 22460905 PMC3320027

[12] Yang W, Soares J, Greninger P, Edelman EJ, Lightfoot H, Forbes S, et al Genomics of Drug Sensitivity in Cancer (GDSC): a resource for therapeutic biomarker discovery in cancer cells. Nucl Acids Res. 2013;41(Database issue):D955–D961. 10.1093/nar/gks1111 23180760 PMC3531057

[13] Kim J, Schoeberl B. Beyond static biomarkers—The dynamic response potential of signaling networks as an alternate biomarker? Sci Signal. 2015;8(408):fs21 10.1126/scisignal.aad4989 26696629

[14] Fey D, Halasz M, Dreidax D, Kennedy SP, Hastings JF, Rauch N, et al Signaling pathway models as biomarkers: Patient-specific simulations of JNK activity predict the survival of neuroblastoma patients. Sci Signal. 2015;8(408). 10.1126/scisignal.aab0990 26696630

[15] Fröhlich F, Kessler T, Weindl D, Shadrin A, Schmiester L, Hache H, et al Efficient parameter estimation enables the prediction of drug response using a mechanistic pan-cancer pathway model. Cell Systems. 2018;7(6):567–579.e6. 10.1016/j.cels.2018.10.013. 30503647

[16] Kanehisa M, Goto S, Furumichi M, Tanabe M, Hirakawa M. KEGG for representation and analysis of molecular networks involving diseases and drugs. Nucleic Acids Res. 2010;38(Database issue):D355–D360. 10.1093/nar/gkp896 19880382 PMC2808910

[17] Croft D, O’Kelly G, Wu G, Haw R, Gillespie M, Matthews L, et al Reactome: A database of reactions, pathways and biological processes. Nucleic Acids Res. 2011;39(Database issue):D691–7. 10.1093/nar/gkq1018 21067998 PMC3013646

[18] Li C, Donizelli M, Rodriguez N, Dharuri H, Endler L, Chelliah V, et al BioModels Database: An enhanced, curated and annotated resource for published quantitative kinetic models. BMC Syst Biol. 2010;4:92 10.1186/1752-0509-4-92 20587024 PMC2909940

[19] Sasagawa S, Ozaki Yi, Fujita K, Kuroda S. Prediction and validation of the distinct dynamics of transient and sustained ERK activation. Nat Cell Biol. 2005;7:365–373. 10.1038/ncb1233 15793571

[20] Schöberl B, Pace EA, Fitzgerald JB, Harms BD, Xu L, Nie L, et al Therapeutically targeting ErbB3: A key node in ligand-induced activation of the ErbB receptor–PI3K axis. Science Signaling. 2009;2(77):ra31. 19567914 10.1126/scisignal.2000352

[21] Fujita KA, Toyoshima Y, Uda S, Ozaki Yi, Kubota H, Kuroda S. Decoupling of receptor and downstream signals in the Akt pathway by its low-pass filter characteristics. Sci Signal. 2010;3(132):ra56 10.1126/scisignal.2000810 20664065

[22] Flobak A, Baudot A, Remy E, Thommesen L, Thieffry D, Kuiper M, et al Discovery of Drug Synergies in Gastric Cancer Cells Predicted by Logical Modeling. PLoS Comput Biol. 2015;. 10.1371/journal.pcbi.1004426 26317215 PMC4567168

[23] Schwarz G. Estimating the dimension of a model. Ann Statist. 1978;6(2):461–464. 10.1214/aos/1176344136

[24] Kneissl J, Keller S, Lorber T, Heindl S, Keller G, Drexler I, et al Association of amphiregulin with the cetuximab sensitivity of gastric cancer cell lines. Int J Oncol. 2012;41(2):733–744. 10.3892/ijo.2012.1479 22614881

[25] den Dunnen J, Dalgleish R, Maglott D, Hart R, Greenblatt M, McGowan-Jordan J, et al HGVS Recommendations for the Description of Sequence Variants: 2016 Update. Hum Mutat. 2016;. 10.1002/humu.22981 26931183

[26] Kang S, Bader AG, PK PKV. Phosphatidylinositol 3-kinase mutations identified in human cancer are oncogenic. Proc Natl Acad Sci USA. 2005;102(3):802–807. 10.1073/pnas.0408864102 15647370 PMC545580

[27] Oda K, Matsuoka Y, Funahashi A, Kitano H. A comprehensive pathway map of epidermal growth factor receptor signaling. Mol Syst Biol. 2005;1(2005.0010). 10.1038/msb4100014 16729045 PMC1681468

[28] Wells A. EGF receptor. Int J Biochem Cell Biol. 1999;31(6):637–643. 10.1016/s1357-2725(99)00015-1 10404636

[29] Hrustanovic G, Lee BJ, Bivona TG. Mechanisms of resistance to EGFR targeted therapies. Cancer Biol Ther. 2013;14(4):304–314. 10.4161/cbt.23627 23358468 PMC3667869

[30] Zhao B, Wang L, Qiu H, Zhang M, Sun L, Peng P, et al Mechanisms of resistance to anti-EGFR therapy in colorectal cancer. Oncotarget. 2017;8(3):3980–4000. 10.18632/oncotarget.14012 28002810 PMC5354808

[31] Raue A, Schilling M, Bachmann J, Matteson A, Schelke M, Kaschek D, et al Lessons learned from quantitative dynamical modeling in systems biology. PLoS ONE. 2013;8(9):e74335 10.1371/journal.pone.0074335 24098642 PMC3787051

[32] Raue A, Steiert B, Schelker M, Kreutz C, Maiwald T, Hass H, et al Data2Dynamics: a modeling environment tailored to parameter estimation in dynamical systems. Bioinformatics. 2015;31(21):3558–3560. 10.1093/bioinformatics/btv405 26142188

[33] Hross S, Hasenauer J. Analysis of CFSE time-series data using division-, age- and label-structured population models. Bioinformatics. 2016;32(15):2321–2329. 10.1093/bioinformatics/btw131 27153577

[34] Steiert B, Timmer J, Kreutz C. L1 regularization facilitates detection of cell type-specific parameters in dynamical systems. Bioinformatics. 2016;32(17):i718–i726. 10.1093/bioinformatics/btw461 27587694 PMC5013918

[35] Heindl S, Eggenstein E, Keller S, Kneissl J, Keller G, Mutze K, et al Relevance of MET activation and genetic alterations of KRAS and E-cadherin for cetuximab sensitivity of gastric cancer cell lines. J Cancer Res Clin Oncol. 2012;. 10.1007/s00432-011-1128-4 PMC11942612 22290393

[36] Lake D, Correa S, J M. Negative feedback regulation of the ERK1/2 MAPK pathway. Cellular and Molecular Life Sciences. 2016;. 10.1007/s00018-016-2297-8 27342992 PMC5075022

[37] Gutenkunst RN, Waterfall JJ, Casey FP, Brown KS, Myers CR, Sethna JP. Universally sloppy parameter sensitivities in systems biology models. PLoS Comput Biol. 2007;3(10):1871–1878. 10.1371/journal.pcbi.0030189 17922568 PMC2000971

[38] Park CH, Cho SY, Ha JD, Jung H, Kim HR, Lee CO, et al Novel c-Met inhibitor suppresses the growth of c-Met-addicted gastric cancer cells. BMC Cancer. 2016;. 10.1186/s12885-016-2058-y PMC4722623 26801760

[39] Xu JM, Wang Y, Wang YL, Wang Y, Liu T, Ni M, et al PIK3CA Mutations Contribute to Acquired Cetuximab Resistance in Patients with Metastatic Colorectal Cancer. Clin Cancer Res. 2017;23(16):4602–4616. 10.1158/1078-0432.CCR-16-2738 28424201 PMC5559326

[40] Harada K, Baba Y, Shigaki H, Ishimoto T, Miyake K, Kosumi K, et al Prognostic and clinical impact of PIK3CA mutation in gastric cancer: pyrosequencing technology and literature review. BMC Cancer. 2016;16:400 10.1186/s12885-016-2422-y 27388016 PMC4936296

[41] Pilotto S, Gkountakos A, Carbognin L, Scarpa A, Tortora G, Bria E. MET exon 14 juxtamembrane splicing mutations: clinical and therapeutical perspectives for cancer therapy. Ann Transl Med. 2017;5(1):2 10.21037/atm.2016.12.33 28164087 PMC5253296

[42] Zhang L, Yang J, Cai J, Song X, Deng J, Huang X, et al A subset of gastric cancers with EGFR amplification and overexpression respond to cetuximab therapy. Sci Rep. 2013;3:2992 10.1038/srep02992 24141978 PMC3801116

[43] Wilson KJ, Gilmore JL, Foley J, Lemmon MA, Riese DJ 2nd. Functional selectivity of EGF family peptide growth factors: implications for cancer. Pharmacol Ther. 2009;122(1):1–8. 10.1016/j.pharmthera.2008.11.008 19135477 PMC2665203

[44] Macdonald-Obermann JL, Pike LJ. Different epidermal growth factor (EGF) receptor ligands show distinct kinetics and biased or partial agonism for homodimer and heterodimer formation. J Biol Chem. 2014;289(38):26178–26188. 10.1074/jbc.M114.586826 25086039 PMC4176247

[45] Roepstorff K, Grandal M, Henriksen L, Knudsen S, Lerdrup M, Grovdal L, et al Differential effects of EGFR ligands on endocytic sorting of the receptor. Traffic. 2009;. 10.1111/j.1600-0854.2009.00943.x 19531065 PMC2723868

[46] Kuperstein I, Bonnet E, Nguyen HA, Cohen D, Viara E, Grieco L, et al Atlas of Cancer Signalling Network: A systems biology resource for integrative analysis of cancer data with Google Maps. Oncogenesis. 2015;4:e160 10.1038/oncsis.2015.19 26192618 PMC4521180

[47] Schneider CA, Rasband WS, Eliceiri KW. NIH Image to ImageJ: 25 years of image analysis. Nature methods. 2012;. 10.1038/nmeth.2089 22930834 PMC5554542

[48] Villaverde AF, Froehlich F, Weindl D, Hasenauer J, Banga JR. Benchmarking optimization methods for parameter estimation in large kinetic models. Bioinformatics. 2018; p. bty736. 10.1093/bioinformatics/bty736 PMC6394396 30816929

[49] Hass H, Loos C, Raimúndez-Álvarez E, Timmer J, Hasenauer J, Kreutz C. Benchmark Problems for Dynamic Modeling of Intracellular Processes. Bioinformatics. 2019; p. btz020. 10.1093/bioinformatics/btz020 PMC6735869 30624608

[50] Kreutz C. New Concepts for Evaluating the Performance of Computational Methods. IFAC-PapersOnLine. 2016;49(26):63–70. 10.1016/j.ifacol.2016.12.104

[51] Burnham KP, Anderson DR. Model selection and multimodel inference: A practical information-theoretic approach. 2nd ed New York, NY: Springer; 2002.

[52] Kreutz C, Raue A, Kaschek D, Timmer J. Profile likelihood in systems biology. FEBS J. 2013;280(11):2564–2571. 10.1111/febs.12276 23581573

[53] Stapor P, Weindl D, Ballnus B, Hug S, Loos C, Fiedler A, et al PESTO: Parameter EStimation TOolbox. Bioinformatics. 2018;34(4):705–707. 10.1093/bioinformatics/btx676 29069312 PMC5860618

[54] Loos C, Krause S, Hasenauer J. Hierarchical optimization for the efficient parametrization of ODE models. Bioinformatics. 2018;34(24):4266–4273. 10.1093/bioinformatics/bty514 30010716 PMC6289139

