# Supplementary figures and images for: Model-based analysis of response and resistance factors of cetuximab treatment in gastric cancer cell lines
Source: PLoS Comput Biol. 2020 Mar 2;16(3):e1007147. doi: 10.1371/journal.pcbi.1007147 (PMC7067490; doi:10.1371/journal.pcbi.1007147)

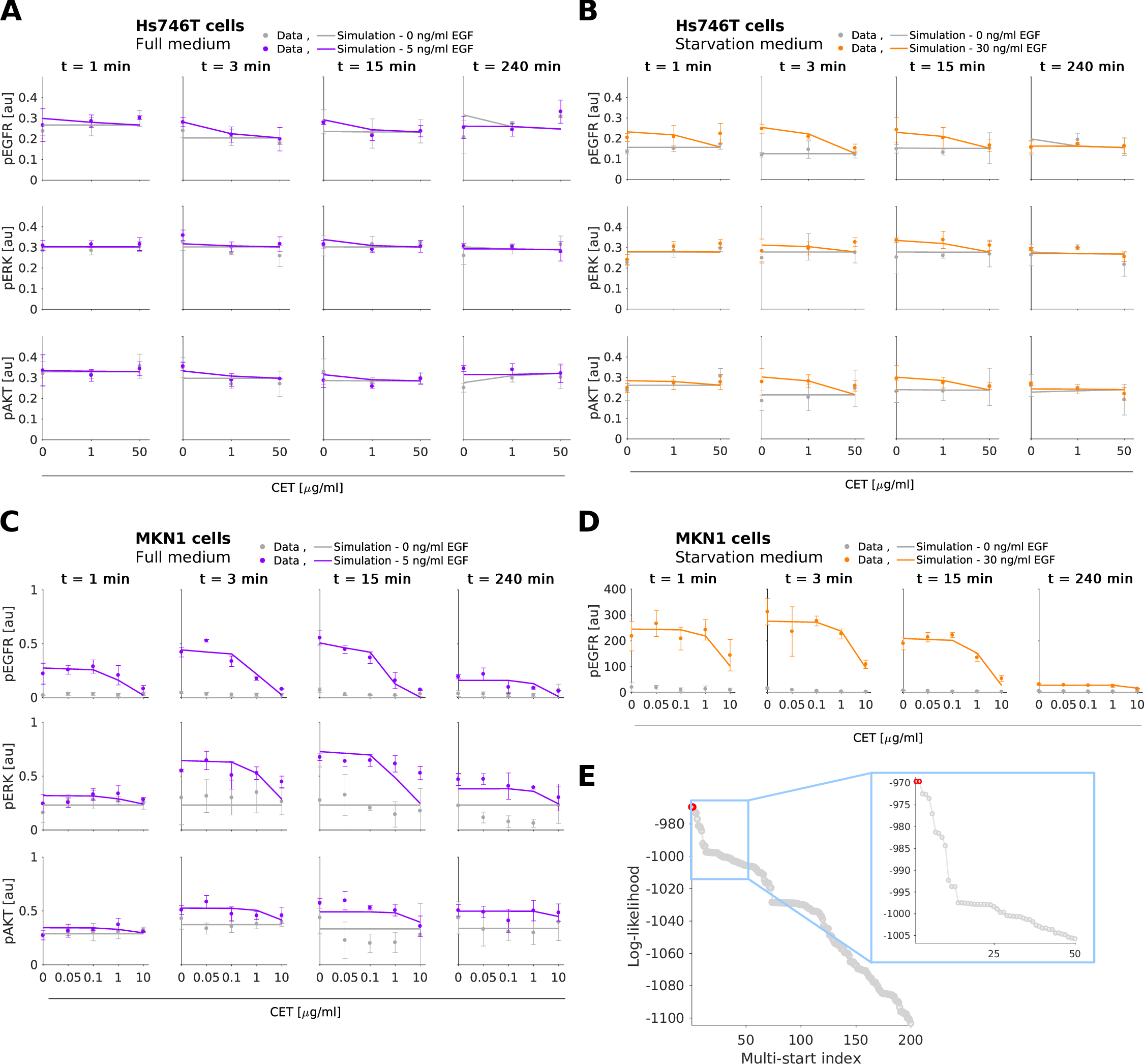

Supplement: S1 Fig — A-B: Comparison of selected experimental data and model fit for Hs746T cell line. C-D: Comparison of selected experimental data and model fit for MKN1 cell line. A-D: Time and dose response data obtained using immunoblotting indicate the mean and standard deviation of three biological experiments. Experimental measurements were scaled to model simulation using the estimated scaling factors. E: Waterfall plots for multi-start local optimization. The best 200 out of 1000 runs are depicted from which a magnification of the best 50 multi-starts is indicated by the blue box. Red dots denote the starts converged to the global optimum within a small numerical margin. Additional data and model fits are provided in S5 and S6 Figs. (TIF) [file pcbi.1007147.s001.tif]

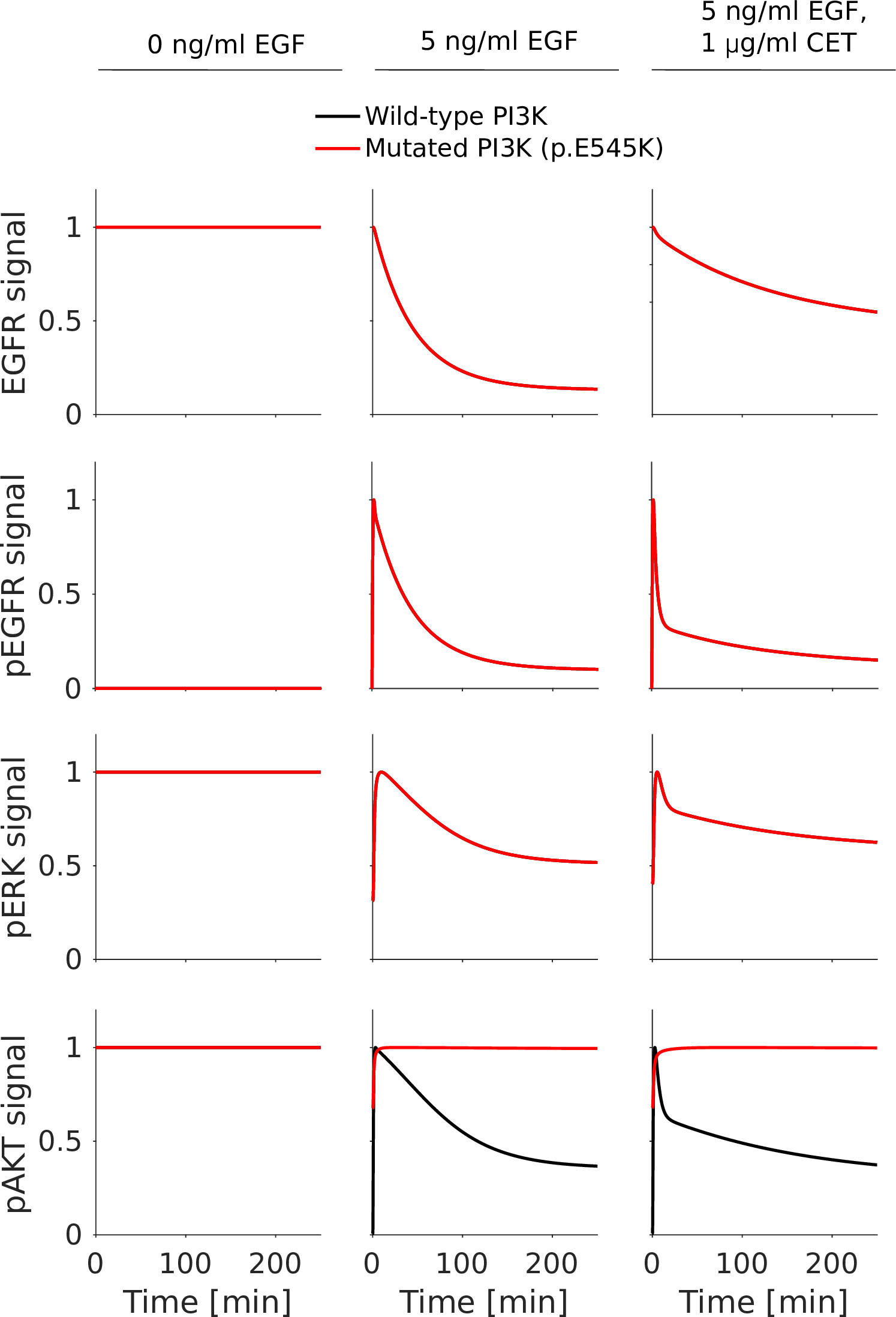

Supplement: S2 Fig — Model prediction of time response of wild-type PI3K (black line) shows a reduction in AKT activity compared to PI3K p.E545K (red line), which remains insensitive. The signal is normalized with respect to the maximum activity level for each observed component. (TIF) [file pcbi.1007147.s002.tif]

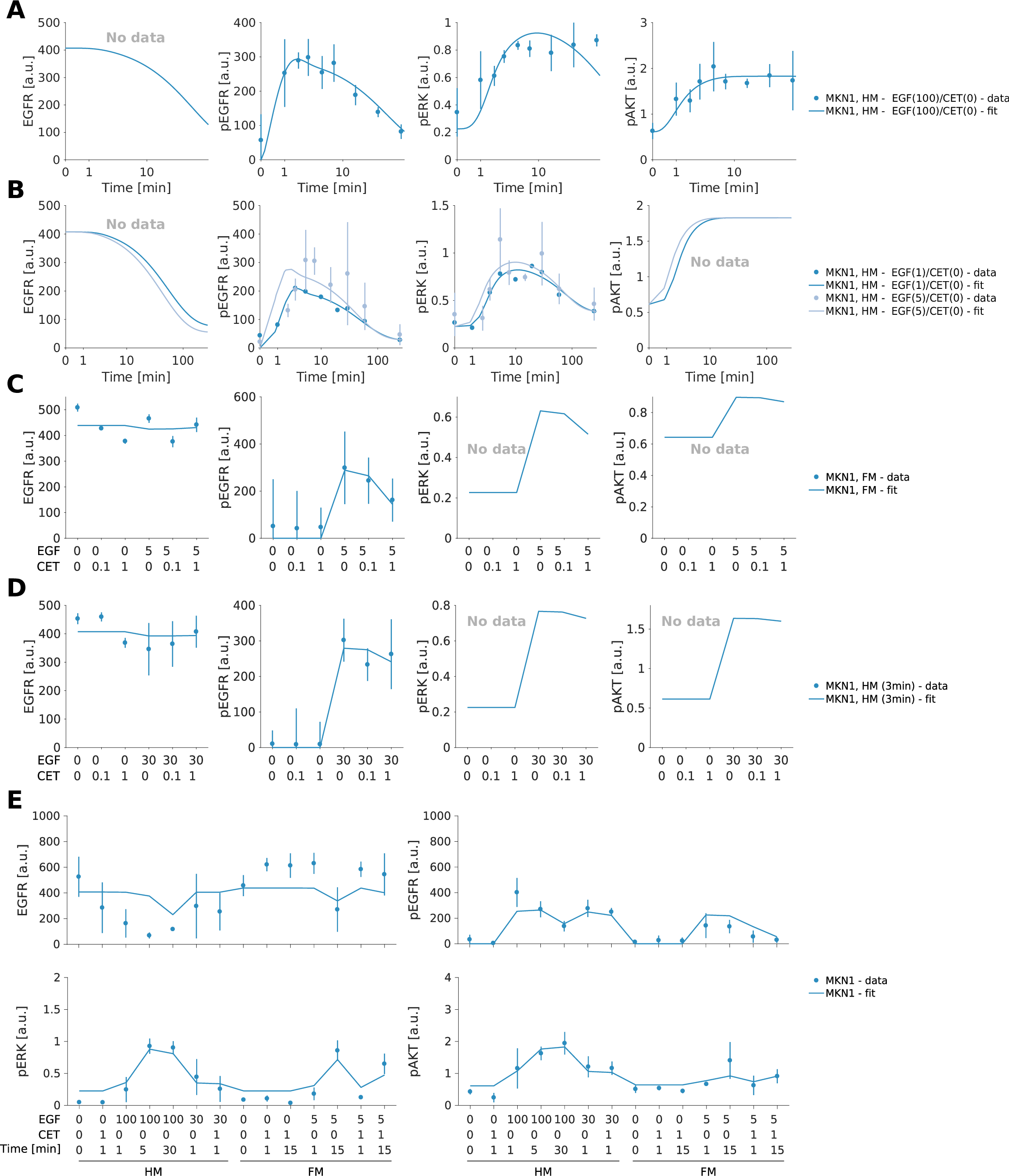

Supplement: S3 Fig — A-B: Time response to different EGF concentrations in starvation culture media (HM). C: Dose response to EGF and cetuximab stimulation at 3 min in rich culture media (FM). D: Dose response to EGF and cetuximab stimulation at 3 min in starvation culture media (HM). E: Dose response to EGF and cetuximab stimulation at 0, 1, 15 and 30 min in full (FM) and starvation culture media (HM). C-E: Specific EGF and cetuximab concentrations are shown along the X axis. (TIF) [file pcbi.1007147.s003.tif]

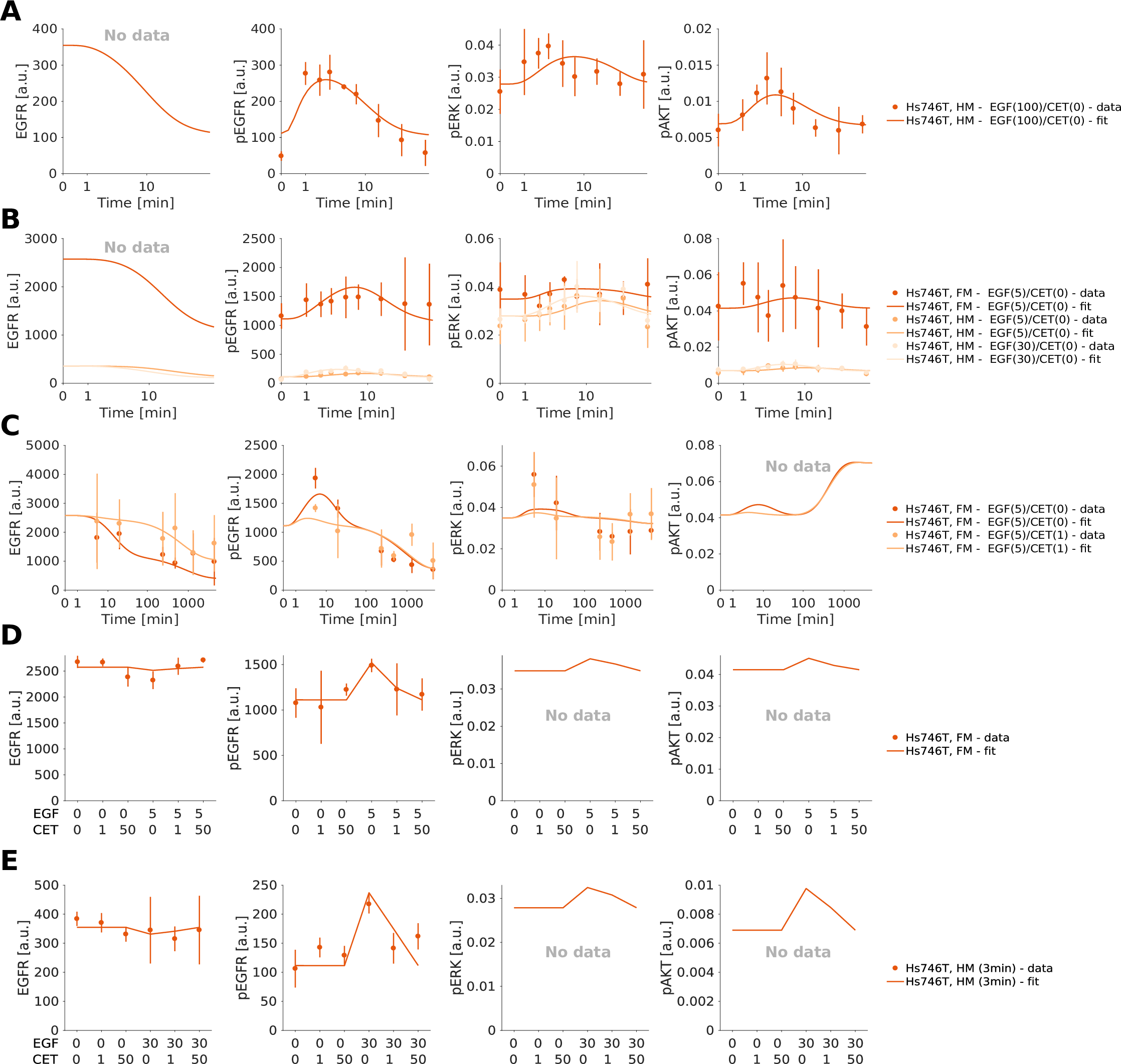

Supplement: S4 Fig — A: Time response to EGF stimulation in starvation culture media (HM). B: Time response to EGF stimulation in full (FM) and starvation culture media (HM). C: Time response to EGF and cetuximab stimulation in rich culture media (FM). D: Dose response to EGF and cetuximab stimulation at 3 min in rich culture media (FM). E: Dose response to EGF and cetuximab stimulation at 3 min in starvation culture media (HM). D-E: Specific EGF and cetuximab concentrations are shown along the X axis. (TIF) [file pcbi.1007147.s004.tif]

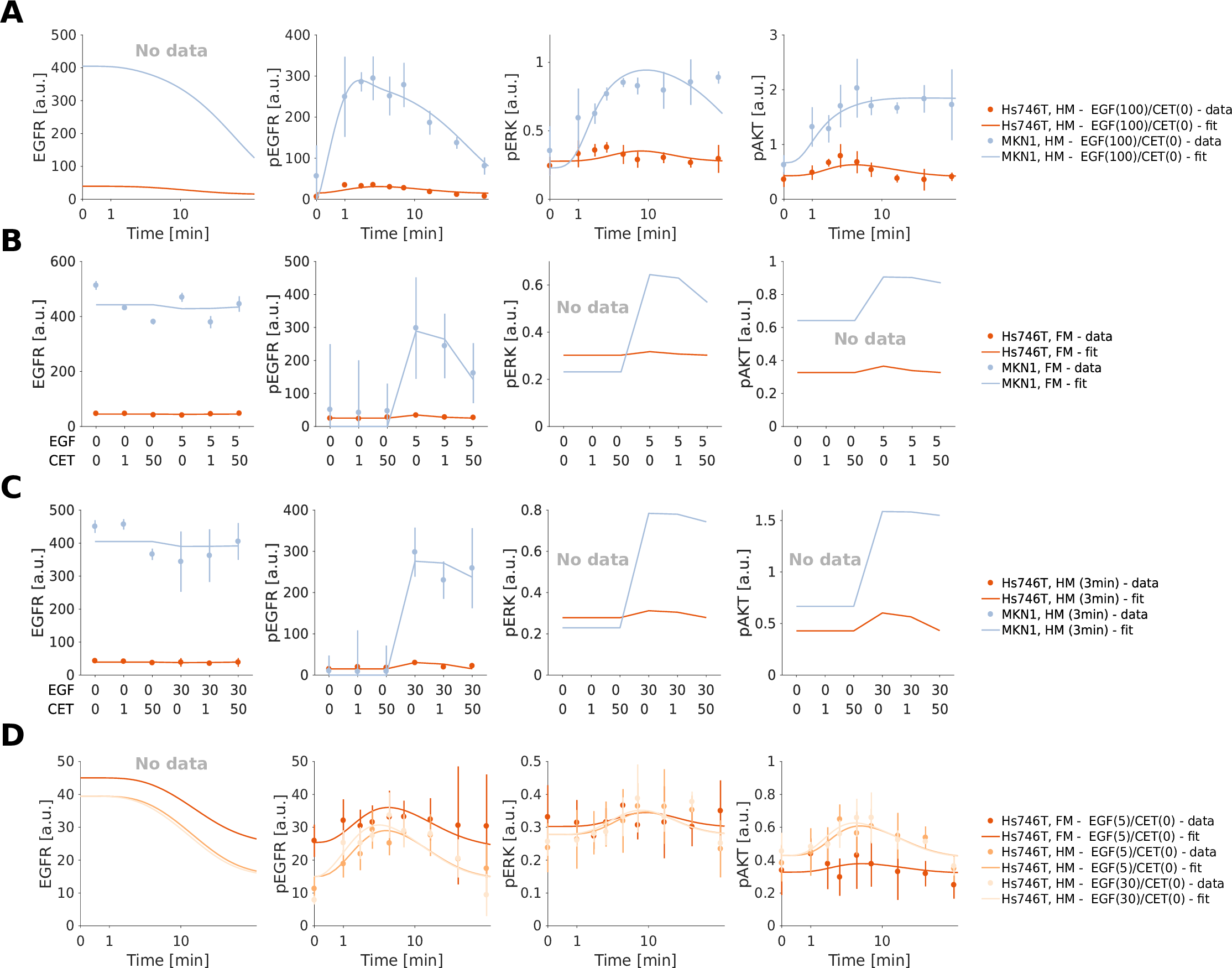

Supplement: S5 Fig — Model fits for the best model (M5). A: Time response to EGF stimulation in starvation culture media (HM). B: Dose response to EGF and cetuximab stimulation at 3 min in rich culture media (FM). C: Dose response to EGF and cetuximab stimulation at 3 min in starvation culture media (HM). D: Time response to EGF stimulation of Hs746T cells in full (FM) and starvation culture media (HM). A-C: Experimental data for both cell lines. B-C: Specific EGF and cetuximab concentrations are shown along the X axis. (TIF) [file pcbi.1007147.s005.tif]

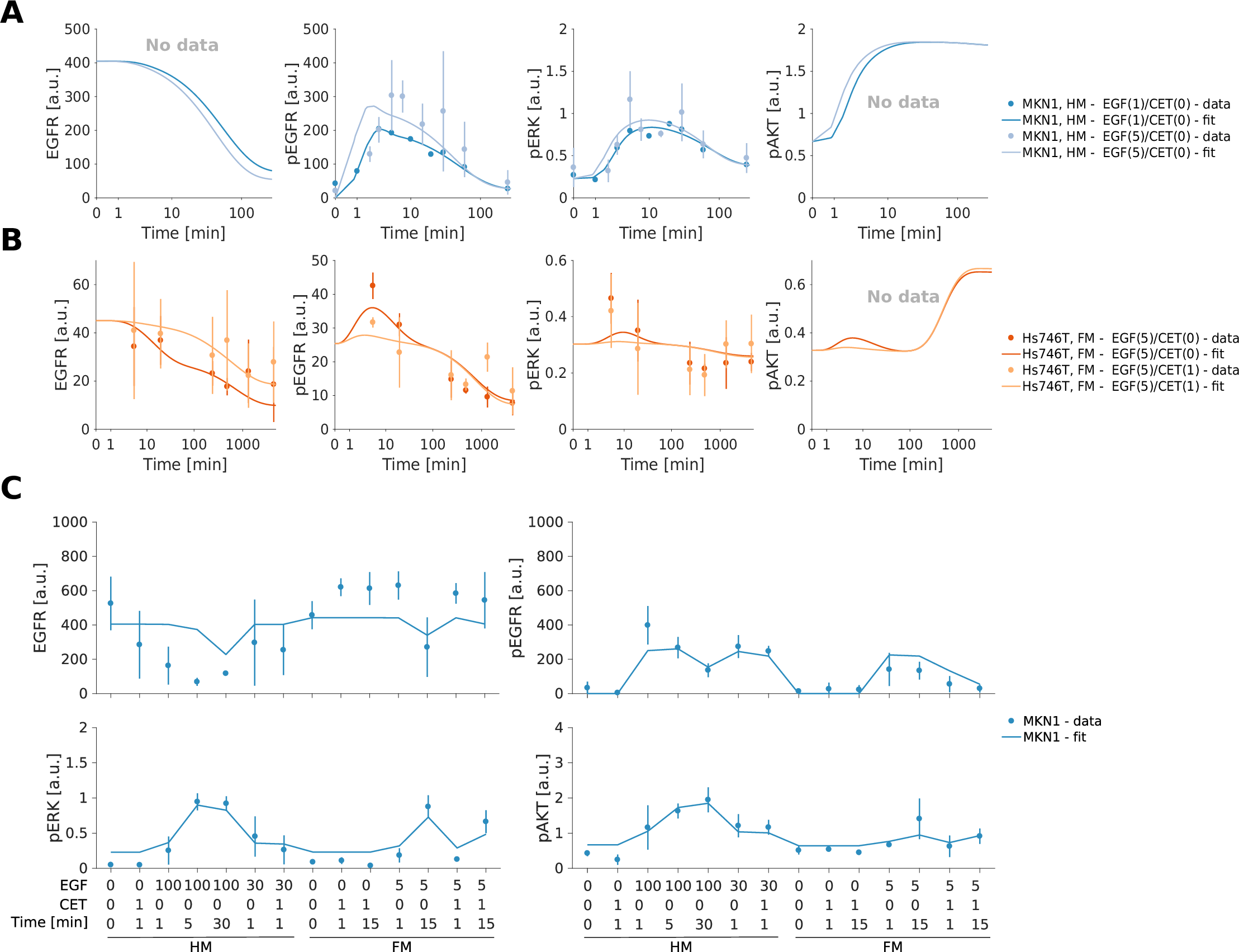

Supplement: S6 Fig — Model fits for the best model (M5). A: Time response to EGF and cetuximab stimulation of MKN1 cells in starvation culture media (HM). B: Time response to EGF and cetuximab stimulation of Hs746T cells in rich culture media (FM). C: Dose response to EGF and cetuximab stimulation at 0, 1, 15 and 30 min of MKN1 cells in rich (FM) and starvation culture media (HM). Specific EGF and cetuximab concentrations, time points and culture media, are shown along the X axis. (TIF) [file pcbi.1007147.s006.tif]

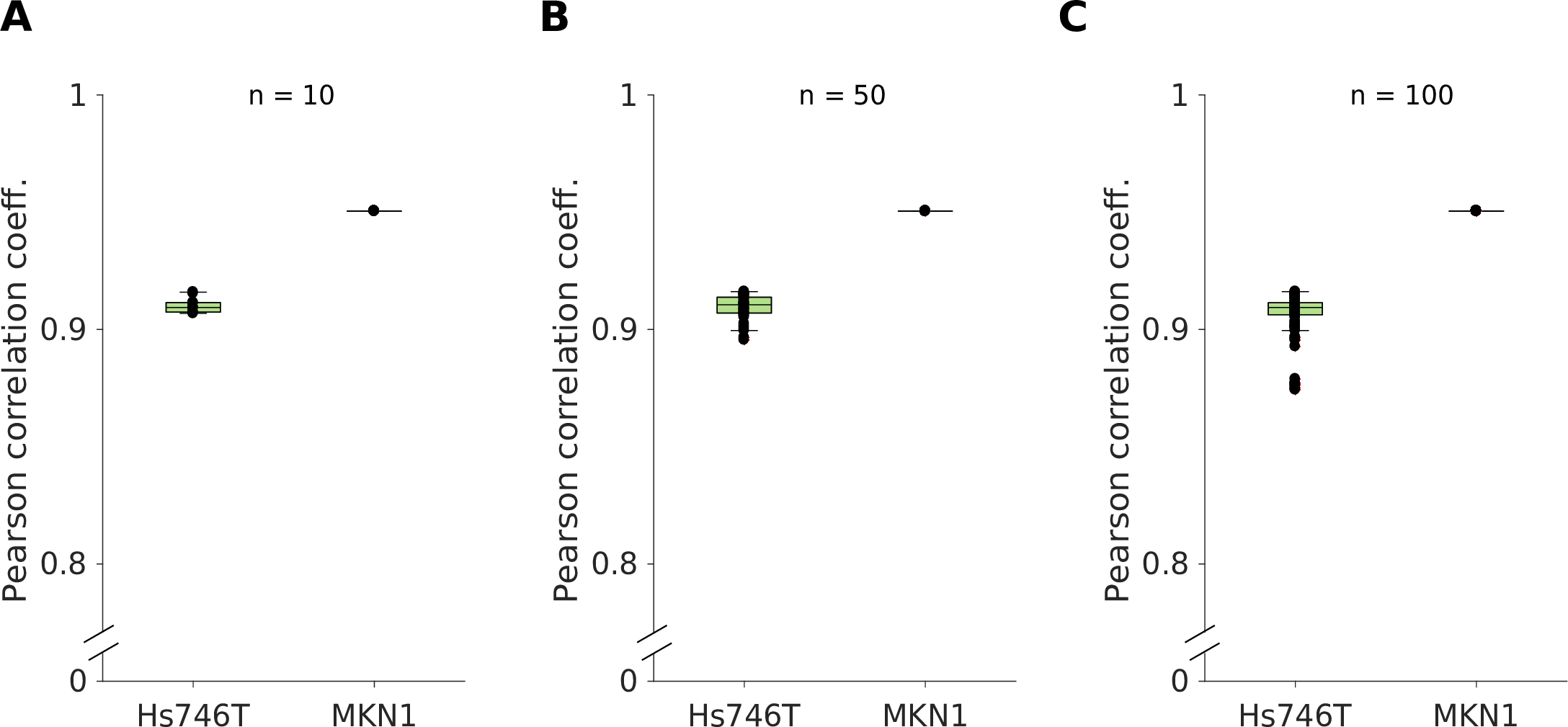

Supplement: S7 Fig — Boxplots for the overall agreement of experimental data and model fits for, A: the best 10 parameter sets, B: the best 50 parameter sets, and C: the best 100 parameter sets. The individual model fits for Hs746T and MKN1 cells are shown. (TIF) [file pcbi.1007147.s007.tif]

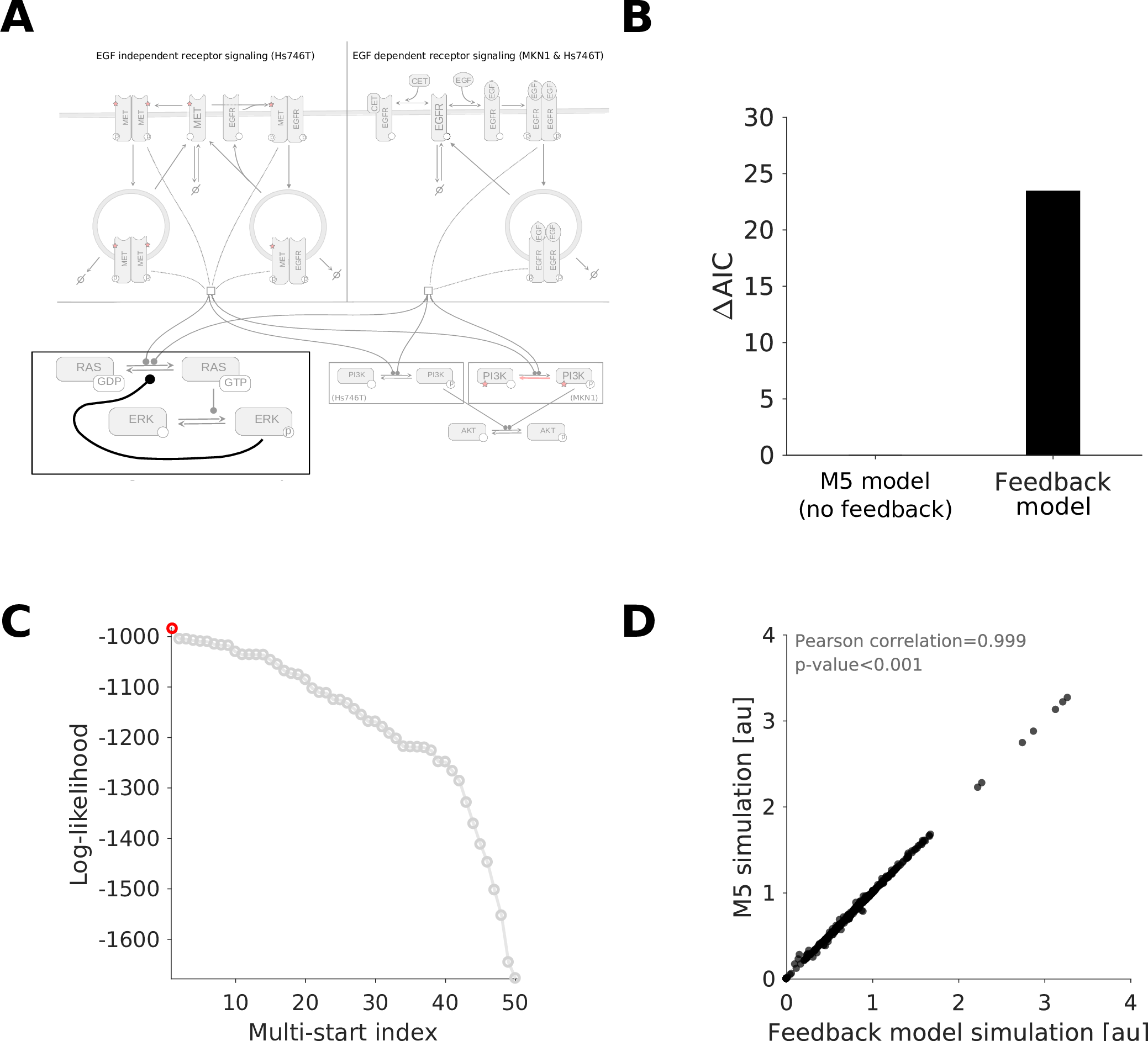

Supplement: S8 Fig — A: Schematic of model including negative feedback regulation from ERK to RAS. B: Differences of AIC values for the model and the best AIC. The parameter estimation results for both models were obtained using 300 local optimization runs. The analysis suggested that the model without feedback is more consistent with the experimental data. C: Waterfall plot for multi-start local optimization. The best 50 out of 300 runs are depicted. Red dots denote the starts converged to the global optimum within a small numerical margin. D: Correlation of simulation results for model M5 (optimal model without feedback) and the model with feedback. (TIF) [file pcbi.1007147.s008.tif]

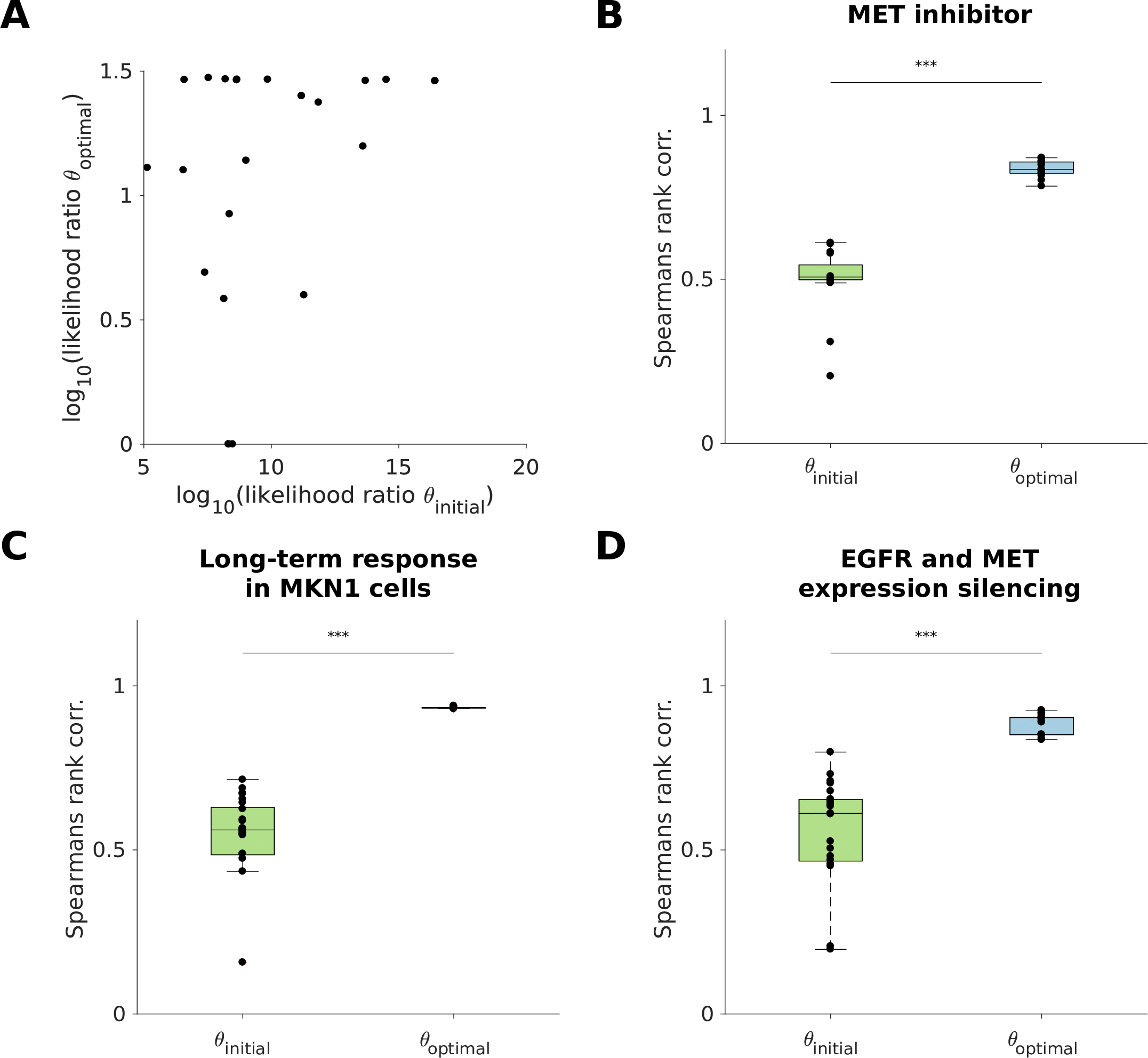

Supplement: S9 Fig — A: Scatter plot of the likelihood ratios for the initial parameter guesses, θinitial, and their corresponding final optimized values, θoptimal. The likelihood ratio was calculated with respect to the best/maximum likelihood estimate found during the multi-start local optimization. The 20 best parameter sets are shown paired with their initial guesses. B-D: Boxplots of the agreement between the validation data and model simulation for the initial parameter guesses, θinitial, and their corresponding final optimized values, θoptimal. The agreement is shown in terms of Spearman’s correlation coefficients. The 20 best parameter sets are shown paired with their initial guesses. The validation datasets shown are (B) MET inhibition, (C) long-term kinetic response in MKN1 cells, and (D) silencing of EGFR and MET expression. (TIF) [file pcbi.1007147.s009.tif]

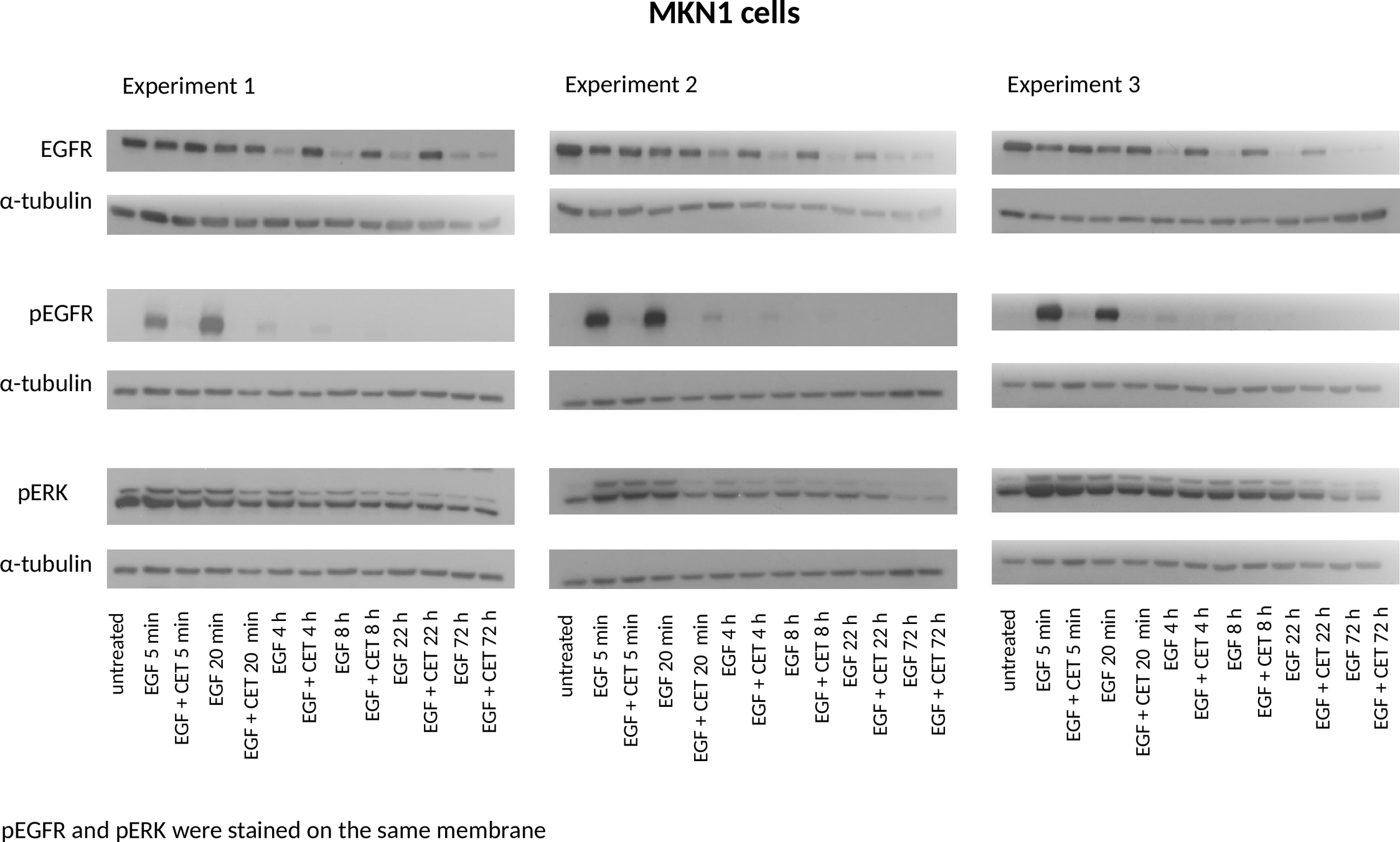

Supplement: S10 Fig — Related to Fig 5D in the main manuscript. (TIF) [file pcbi.1007147.s010.tif]

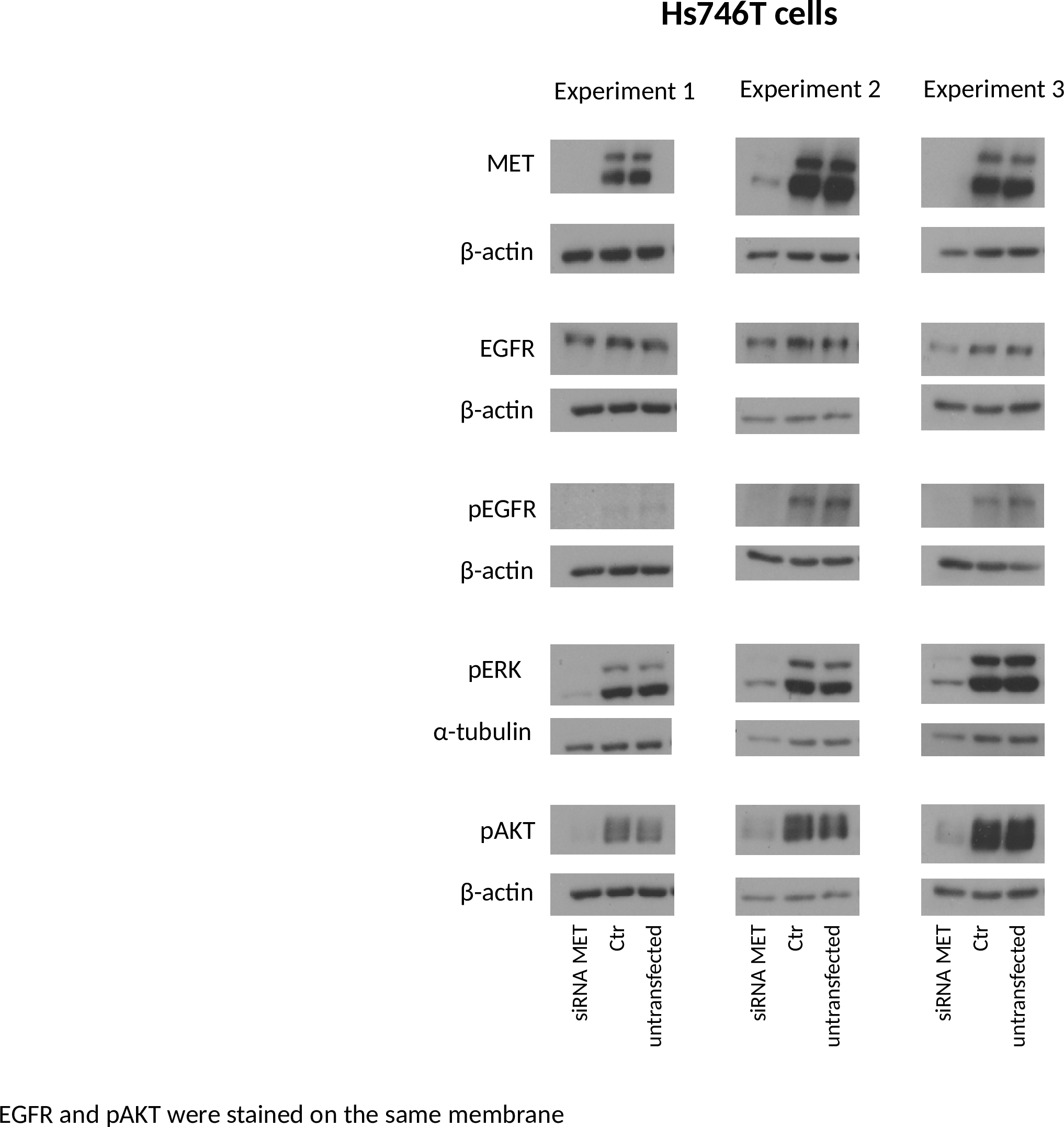

Supplement: S11 Fig — Related to Fig 6C in the main manuscript. (TIF) [file pcbi.1007147.s011.tif]

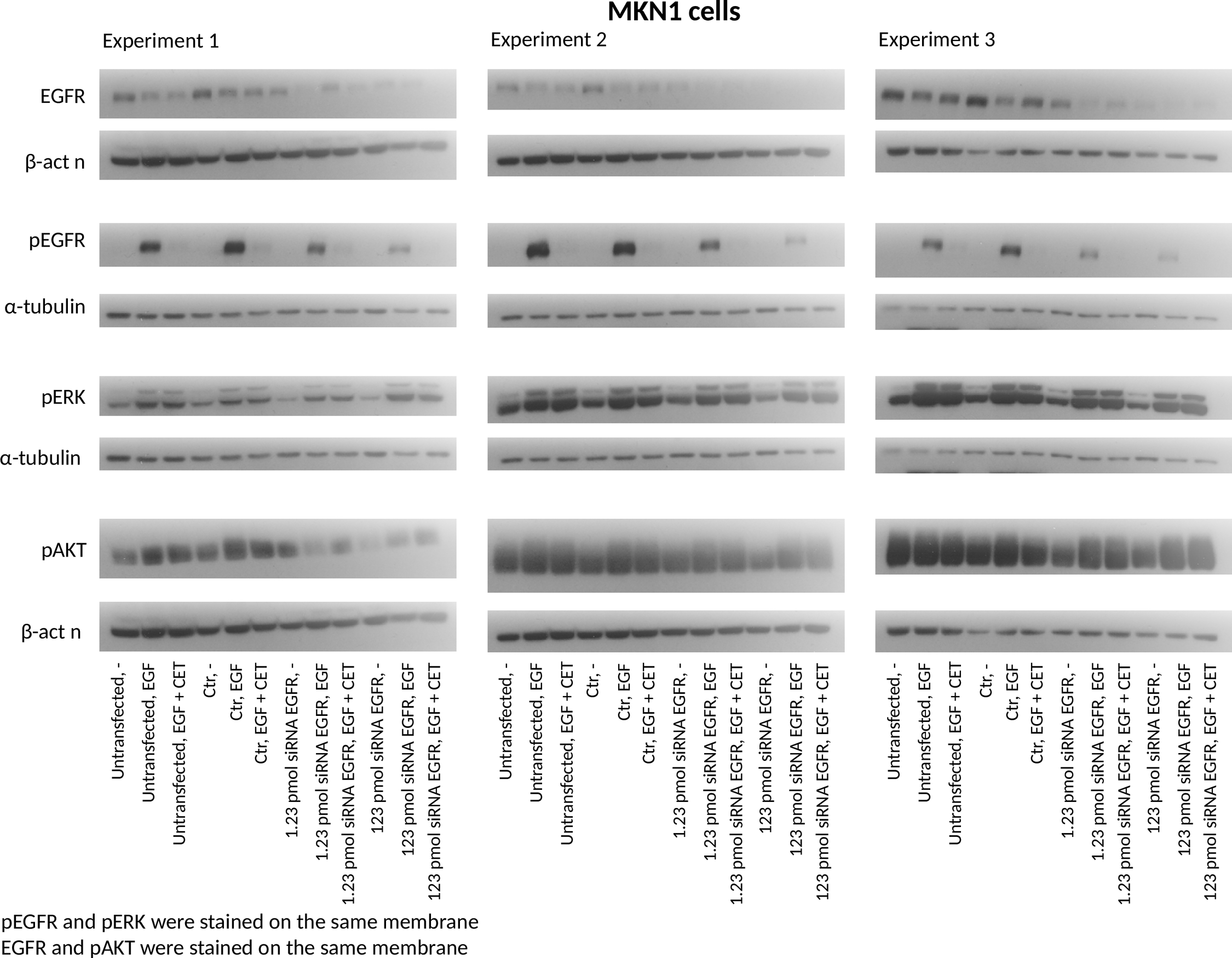

Supplement: S12 Fig — Related to Fig 6E in the main manuscript. (TIF) [file pcbi.1007147.s012.tif]
