# Supplementary material for: Model-based analysis of response and resistance factors of cetuximab treatment in gastric cancer cell lines
Source: PLoS Comput Biol. 2020 Mar 2;16(3):e1007147. doi: 10.1371/journal.pcbi.1007147 (PMC7067490; doi:10.1371/journal.pcbi.1007147)
Supplement: S1 Text — (PDF) [file pcbi.1007147.s013.pdf]

Supplementary Material

Model-based analysis of response and resistance factors of cetuximab treatment in gastric cancer cell lines

Elba Raimúndez [1,2], Simone Keller [3], Gwen Zwingenberger [3], Karolin Ebert [3], Sabine Hug [1,2], Fabian J. Theis [1,2], Dieter Maier [4], Birgit Luber [3], and Jan Hasenauer [1,2,5*]

[1] Helmholtz Zentrum München - German Research Center for Environmental Health, Institute of Computational Biology, Neuherberg 85764, Germany

[2] Technische Universität München, Center for Mathematics, Chair of Mathematical Modeling of Biological Systems, Garching 85748, Germany

[3] Technical University of Munich, School of Medicine, Klinikum rechts der Isar, Institute of Pathology, 81675 München, Germany

[4] Biomax Informatics AG, Planegg

[5] Faculty of Mathematics and Natural Sciences, University of Bonn, 53113 Bonn, Germany

[*] To whom correspondence should be addressed

Contents

1 Data pre-processing **2**

2 Model fits to experimental data **2**

3 Model reactions **2**

4 Candidate reactions for cell line specificity **5**

5 Model and model parameters **5**

6 Optimization settings **6**

7 Additional model structures **6**

8 Validation of the parameter sets **6**

9 Experimental data for model validation **7**

1 Data pre-processing

In this study, we consider a comprehensive collection of Western blot data. As only intensity values on a single gel are comparable, the mapping of the data to the model simulation requires scaling factors (see Loos et al. [2018] for a detailed discussion). Yet, due to the large number of Western blots, more than one hundred scaling factors would be necessary. This increases the dimensionality of the parameter estimation problem and can cause convergence problems. The problem can be eliminated using novel hierarchical optimization methods [Loos et al., 2018], however, this approach is not supported by `Data2Dynamics` – the modeling toolbox we used for this study.

To reduce the number of scaling parameters, we pre-processed the experimental data by aligning replicates. Replicates are in this case the experimental data for different Western blots capturing the same experimental conditions. Mathematically, we consider the experimental data

$$\bar{y}_{r,i} = s_r(\mu_i + \epsilon_{r,i}), \qquad \epsilon_{r,i} \sim \mathcal{N}(0, \sigma^2)$$

with $r = 1, \ldots, R$ indexing the replicate and $i = 1, \ldots, I$ indexing the experimental condition. The experimental condition is a combination of cell line, treatment and time point. The true level of a protein of phospho-protein in experimental condition i is denoted by μ_i, and corrupted by measurement noise $\epsilon_{r,i}$. The scaling factor associated to different replicates (or Western blots) are denoted by s_r. As $\bar{y}_{r_1,i}$ and $\bar{y}_{r_2,i}$ should agree up to measurement noise and scaling factor, we inferred estimates for all but one scaling factor by solving the optimization problem

$$\underset{s_2,\ldots,s_I,\sigma}{\operatorname{argmin}} \left\{ -\log(\mathcal{L}) := \frac{1}{2} \sum_i \log(2\pi\sigma^2) \left(\frac{\frac{1}{s_r}\bar{y}_{r,i} - \hat{\mu}_i}{\sigma} \right)^2 \right\},$$

$$\text{subject to} \quad \hat{\mu}_i = \sum_r \frac{1}{s_r} \bar{y}_{r,i}, \quad i = 1, \ldots, I$$

with $s_1 = 1$ for $r = 1$. The result of the optimization are the pre-processed data $\hat{\mu}_i$, $i = 1, \ldots, I$ and the estimated standard deviation σ. The pre-processed data $\hat{\mu}_i$ aggregated the information of the replicates by compensating for the individual scales. Without lost of generality, the scale of the first replicate was considered as reference ($s_1 = 1$), hence, $\mu_i \approx s_1\hat{\mu}_i$ (up to measurement noise).

The pre-processing reduced the number of scaling factors for an experimental dataset from R to 1 and renders the optimization problem more tractable. This scaling factor effectively corresponds to the scaling factor for the first replicate s_1.

2 Model fits to experimental data

In the main manuscript parts of the large experimental dataset and the corresponding model simulations for the individual fitting of the cell line MKN1 (Fig 2), the individual fitting of the cell line Hs746T (Fig 3), and the combined fitting of MKN1 and Hs746T for the best model (M5) (Fig 5 and Fig S1) are shown. The large datasets were complemented by smaller and more unstructured datasets. These datasets and the corresponding model simulations are depicted in Fig S3, S4, S5 and S6.

3 Model reactions

In the main manuscript a distinction between single reactions describing multi-step signaling processes and describing direct reactions is introduced, which is then used to identify cell line specific reaction rates (Fig 4). In this section, the biochemical reactions implemented in the proposed pathway model are reported. The parts that are exclusive for Hs746T

cells are MMET receptor dynamics and EGFR:MMET heterodimer dynamics. Additionally, PI3K is only expressed in Hs746T cells, while MPI3K only in MKN1 cells. The remaining reactions and biochemical species are common for both cell lines. All model reactions follow mass action kinetics. For a more detailed description – including reaction rates – refer to the SBML model.

EGF receptor dynamics

R1: $\emptyset \xrightarrow{1}$ EGFR
R2: EGFR $\rightarrow \emptyset$ ($*$, membrane turnover)
R3: EGFR + CET $\rightarrow$ EGFR:CET
R4: EGFR:CET $\rightarrow$ EGFR + CET
R5: EGFR:CET $\rightarrow \emptyset$ ($*$, membrane turnover)
R6: EGFR + EGF $\rightarrow$ EGFR:EGF
R7: EGFR:EGF $\rightarrow$ EGFR + EGF
R8: EGFR:EGF + EGFR:EGF $\rightarrow$ $(\text{EGFR:EGF})_2$
R9: $(\text{EGFR:EGF})_2 \rightarrow (\text{pEGFR:EGF})_2$
R10: $(\text{pEGFR:EGF})_2 \rightarrow (\text{pEGFR:EGF})_{2,\text{endocytic vesicle}}$ ($*$)
R11: $(\text{pEGFR:EGF})_{2,\text{endocytic vesicle}} \rightarrow$ EGFR + EGFR ($*$)
R12: EGFR:EGF $\rightarrow \emptyset$ ($*$, membrane turnover)
R13: $(\text{EGFR:EGF})_2 \rightarrow \emptyset$ ($*$, membrane turnover)
R14: $(\text{pEGFR:EGF})_{2,\text{endocytic vesicle}} \rightarrow \emptyset$ ($*$)

This module consists of 14 reactions describing – among other things – synthesis, ligand and cetuximab (CET) binding, dimerization, internalization and degradation of EGFR. In total 7 out of 14 reactions summarize multi-step processes, denoted by ($*$). For the dynamics of R2, R5, R12 and R13, we assumed a common kinetic rate describing the membrane turnover. Therefore, from these 7 reactions, 4 different kinetic rates were considered as candidates for cell-line specificity.

[1] For the EGFR silencing experiments the synthesis rate of EGFR is multiplied with the fold-change observed after siRNA treatment (see Fig S12).

MMET receptor dynamics

R15: $\emptyset \xrightarrow{2}$ MMET
R16: MMET $\rightarrow \emptyset$
R17: MMET + inhibitor $\xrightarrow{3}$ MMET:inhibitor
R18: MMET:inhibitor $\rightarrow$ MMET + inhibitor
R19: MMET:inhibitor $\rightarrow \emptyset$
R20: MMET + MMET $\rightarrow$ MMET_2
R21: $\text{MMET}_2 \rightarrow \text{pMMET}_2$
R22: $\text{pMMET}_2 \rightarrow \text{pMMET}_{2,\text{endocytic vesicle}}$
R23: $\text{pMMET}_{2,\text{endocytic vesicle}} \rightarrow$ MMET + MMET
R24: $\text{MMET}_2 \rightarrow \emptyset$
R25: $\text{pMMET}_{2,\text{endocytic vesicle}} \rightarrow \emptyset$
R26: MMET + MMET:inhibitor $\rightarrow$ MMET_2:inhibitor
R27: MMET:inhibitor + MMET:inhibitor $\rightarrow$ $(\text{MMET:inhibitor})_2$
R28: MMET_2 + inhibitor $\xrightarrow{3}$ MMET_2:inhibitor
R29: MMET_2:inhibitor + inhibitor $\xrightarrow{3}$ $(\text{MMET:METinh})_2$
R30: MMET_2:inhibitor $\rightarrow \emptyset$
R31: $(\text{MMET:inhibitor})_2 \rightarrow \emptyset$

This module consists of 16 reactions describing – among other things – synthesis, ligand and MMET inhibitor binding, dimerization, internalization and degradation of MMET. This signaling module is specific for Hs746T cells since MMET is not expressed in MKN1 cells. Consequently, we excluded this group of reactions from the statistical analysis presented in the main manuscript (Fig 4) and, therefore, we did not highlight the different reaction types.

[2] For the MET silencing experiments the synthesis rate of MET is multiplied with the fold-change observed after siRNA treatment (see Fig S11).

[3] The K_d value of the MET inhibitor is known from Park et al. [2016] and the inhibitor concentrations are set in the experiments.

EGFR:MMET heterodimer dynamics

R32: MMET + EGFR → MMET:EGFR
R33: MMET:EGFR → pMMET:pEGFR
R34: pMMET:pEGFR → pMMET:$\text{pEGFR}_{\text{endocytic vesicle}}$
R35: pMMET:$\text{pEGFR}_{\text{endocytic vesicle}}$ → MMET + EGFR
R36: MMET:EGFR → ∅
R37: pMMET:$\text{pEGFR}_{\text{endocytic vesicle}}$ → ∅
R38: MMET:inhibitor + EGFR → EGFR:MMET:inhibitor

This module consists of 7 reactions describing the dynamics for the EGFR and MMET heterodimers. This signaling module is specific for Hs746T cells since MMET is not expressed in MKN1 cells. Consequently, we excluded this group of reactions from the statistical analysis presented in the main manuscript (Fig 4) and, therefore, we did not highlight the different reaction types.

RAS-MAPK downstream signaling

R39: RAS-GDP → RAS-GTP (∗)
R40: RAS-GDP + $(\text{pEGFR:EGF})_2$ → RAS-GTP + $(\text{pEGFR:EGF})_2$ (∗, EGFR induced activation)
R41: RAS-GDP + $(\text{pEGFR:EGF})_{2,\text{endocytic vesicle}}$ → RAS-GTP + $(\text{pEGFR:EGF})_{2,\text{endocytic vesicle}}$ (∗, EGFR induced activation)
R42: RAS-GDP + pMMET:pEGFR → RAS-GTP + pMMET:pEGFR
R43: RAS-GDP + pMMET:$\text{pEGFR}_{\text{endocytic vesicle}}$ → RAS-GTP + pMMET:$\text{pEGFR}_{\text{endocytic vesicle}}$
R44: RAS-GDP + pMMET_2 → RAS-GTP + pMMET_2
R45: RAS-GDP + $\text{pMMET}_{2,\text{endocytic vesicle}}$ → RAS-GTP + $\text{pMMET}_{2,\text{endocytic vesicle}}$
R46: RAS-GTP → RAS-GDP (∗)
R47: ERK + RAS-GTP → pERK + RAS-GTP (∗)
R48: pERK → ERK

This module consists of 10 reactions describing – among other things – basal and receptor induced activation (including EGFR, MMET and their association), and downstream signaling of RAS. We excluded the reactions involving the biochemical species pMMET_2 and pMMET:pEGFR – also their corresponding internalized forms – as they are only expressed in Hs746T cells. This leads to a total of 5 out of 10 reactions summarizing multi-step processes, denoted by (∗). For the dynamics of R10 and R41, we assumed a common kinetic rate describing the EGFR induced activation of RAS. Therefore, from these 5 reactions, 4 different kinetic rates were considered as candidates for cell-line specificity.

PI3K- and MPI3K-AKT downstream signaling

R49: PI3K → pPI3K
R50: PI3K + $(\text{pEGFR:EGF})_2$ → pPI3K + $(\text{pEGFR:EGF})_2$
R51: PI3K + $(\text{pEGFR:EGF})_{2,\text{endocytic vesicle}}$ → pPI3K + $(\text{pEGFR:EGF})_{2,\text{endocytic vesicle}}$

R52: PI3K + pMMET:pEGFR $\rightarrow$ pPI3K + pMMET:pEGFR
R53: PI3K + pMMET:pEGFR$_{\text{endocytic vesicle}}$ $\rightarrow$ pPI3K + pMMET:pEGFR$_{\text{endocytic vesicle}}$
R54: PI3K + pMMET$_2$ $\rightarrow$ pPI3K + pMMET$_2$
R55: PI3K + pMMET$_{2,\text{endocytic vesicle}}$ $\rightarrow$ pPI3K + pMMET$_{2,\text{endocytic vesicle}}$
R56: pPI3K $\rightarrow$ PI3K $(*, \dagger)$
R57: MPI3K $\rightarrow$ pMPI3K
R58: MPI3K + (pEGFR:EGF)$_2$ $\rightarrow$ pMPI3K + (pEGFR:EGF)$_2$
R59: MPI3K + (pEGFR:EGF)$_{2,\text{endocytic vesicle}}$ $\rightarrow$ pMPI3K + (pEGFR:EGF)$_{2,\text{endocytic vesicle}}$
R60: MPI3K + pMMET:pEGFR $\rightarrow$ pMPI3K + pMMET:pEGFR
R61: MPI3K + pMMET:pEGFR$_{\text{endocytic vesicle}}$ $\rightarrow$ pMPI3K + pMMET:pEGFR$_{\text{endocytic vesicle}}$
R62: MPI3K + pMMET$_2$ $\rightarrow$ pMPI3K + pMMET$_2$
R63: MPI3K + pMMET$_{2,\text{endocytic vesicle}}$ $\rightarrow$ pMPI3K + pMMET$_{2,\text{endocytic vesicle}}$
R64: pMPI3K $\rightarrow$ MPI3K $(*, \dagger)$
R65: AKT + pPI3K $\rightarrow$ pAKT + pPI3K $(*,$ PI3K/MPI3K induced activation)
R66: AKT + pMPI3K $\rightarrow$ pAKT + pMPI3K $(*,$ PI3K/MPI3K induced activation)
R67: pAKT $\rightarrow$ AKT $(*)$

This module consists of 19 reactions describing – among other things – basal and receptor induced activation, and downstream signaling of PI3K – expressed in Hs746T cells – and MPI3K – expressed in MKN1 cells. All kinetic rates defined for PI3K and MPI3K are common between the cell lines, except for the inactivation rate – due to PI3K mutation in MKN1 cells – indicated by $(\dagger)$. We excluded the reactions involving the biochemical species pMMET$_2$ and pMMET:pEGFR – also their corresponding internalized forms – as they are only expressed in Hs746T cells. This leads to a total of 4 out of 19 reactions summarizing multi-step processes, denoted by $(*)$. We assumed a common kinetic rate describing the PI3K (and MPI3K) induced activation of AKT. Therefore, from these 4 reactions, 3 different kinetic rates were considered as candidates for cell-line specificity.

4 Candidate reactions for cell line specificity

In the main manuscript the identification of cell line specific reaction rates is shown (Fig 4). Here, a detailed description of these reaction rates is provided in Table S1. For simplicity, in the following table we show the biochemical reactions in which the kinetic rates is involved. Note that the same kinetic rate can appear in different reactions, e.g., rate for basal membrane turnover in R2, R5, R12 and R13. For a more detailed description refer to the SBML model.

5 Model and model parameters

The model accounts of 27 biochemical species. Protein abundances for RAS, ERK, PI3K, MPI3K and AKT were assumed to be conserved. This results in an ODE model with 22 state variables. As the absolute abundances of the conserved species are unknown, we consider as state the relative abundance with the concentration in MKN1 cells and rich culture media as reference point for RAS, ERK, MPI3K and AKT. As PI3K is not represented in MKN1 cells, we used as reference point the concentration in Hs746T cells and rich culture media. The changes in expression levels and synthesis between the different culture media and cell lines were estimated as proposed by Steiert et al. [2016].

As initial conditions for the simulations of time and dose response data, we employed the steady state of the unperturbed system. This steady state is specific to the combination of cell line and culture medium.

We screened the literature for the kinetic rates considered in the model. Using the obtained information, we defined prior distributions for individual rate constants. We considered normal distributions of the log-transformed paper values. The normal priors were centered around, i.e. mean, the corresponding literature values with a standard deviation of 0.2. The values are listed in Table S2.

6 Optimization settings

For the parameter optimization of the MKN1 model, Hs746T model and combined model, the MATLAB functions *lsqnonlin* and *fmincon* were considered. For the MKN1 model and the combined model, *lsqnonlin* achieved the better optimization results. For the Hs746T model, *fmincon* achieved the better results. For each model, we used the best results across the optimization algorithms. Based on the optimizer performance observed for the combined model, the model selection was performed using *lsqnonlin*.

For all the calibrated models – regardless of the optimizer – the following optimizer tolerances were used

- maximum number of iterations allowed (*MaxIter*) was set to 10^4,
- maximum number of function evaluations allowed (*MaxFunEvals*) was set to 10^4,
- termination tolerance on the function value (*TolFun*) was set to 10^{-9}, and
- termination tolerance on x (*TolX*) was set to 10^{-10},

and the following ODE solver tolerances were used

- relative tolerance (*rtol*) was set to 10^{-8},
- absolute tolerance (*atol*) was set to 10^{-8}, and
- maximum number of integration steps (*maxsteps*) was set to 10^6.

The remaining settings for optimizer and ODE solver were set to the default values provided by `Data2Dynamics`.

7 Additional model structures

In the main manuscript we present the results corresponding to the model described in the Supplementary Material, Section 3. Here, we evaluate whether the quality of the fit can be substantially improved by including the negative feedback interaction from ERK to RAS [Lake et al., 2016] (Fig S8A). As the negative feedback is mediated by decreasing the activation of RAS, we can model this feedback by the reaction:

$$\text{RAS:GDP} \rightarrow \text{RAS:GTP}, \qquad \text{with rate} \qquad k_a[\text{activated receptor}][\text{RAS:GDP}]\frac{K_i}{(K_i + [\text{pERK}])}.$$

with K_i being the inhibition strength, [activated receptor] being the sum of concentration of $(\text{pEGFR:EGF})_2$, $(\text{pEGFR:EGF})_{2,\text{endocytic vesicle}}$, pMMET:pEGFR, $\text{pMMET:pEGFR}_{\text{endocytic vesicle}}$, pMMET_2 or $\text{pMMET}_{2,\text{endocytic vesicle}}$, and k_a being the activation rate of RAS:GDP by a single activated receptor.

We fitted the extended model using the experimental data presented in the main manuscript (Fig 1C-D). The AIC value of the extended model is higher than for the model without negative feedback (Fig S8B), which suggest that the model without the feedback loop is more consistent with the experimental data.

We note that the difference observed between the AIC values is larger than two times the number of additional parameters. This implies that the model with the feedback loop provides a worst fit than the original model. This can be explained by the fact that (i) the model with feedback is not a special case of the model without feedback (as a rate of zero is not in the admissible set) or (ii) that the multi-start optimization did not find the optimal parameter value (Fig S8C).

8 Validation of the parameter sets

To dissect the contribution of the selected model topology and the estimated parameter values on the model predictions

presented in the main manuscript (Fig 5 and 6), we evaluated the prediction accuracy for random parameters and optimized parameters. As all the model predictions were performed with the same model (named *M5* in the main manuscript), we first looked at the differences in the likelihood value for the 20 best fits compared to the best likelihood estimate (Fig S9A). This already indicates a worst performance of the initial parameter guesses as they show a larger likelihood ratio compared to the final optimal parameters. Additionally, we looked at the correlations between the model predictions and each validation dataset: MET inhibition (Fig S9B), long-term response in MKN1 cells (Fig S9C), and EGFR and MET expression silencing experiments (Fig S9D). We found that already random parameter values achieve a positive correlation of the model predictions and the validation data, implying that there is information in the model topology. However, the correlation is substantially higher fitted parameters, meaning that the parameter estimation improved the predictive power. Accordingly, model structure and estimated model parameter contribute to the observed prediction accuracy.

9 Experimental data for model validation

In Fig 5 and 6 in the main manuscript we present the quantification of Western blots used for model validation. The corresponding images are provided in Fig S10-12.

References

Lake, D., Correa, S., and J, M. (2016). Negative feedback regulation of the erk1/2 mapk pathway. *Cellular and Molecular Life Sciences*.

Loos, C., Krause, S., and Hasenauer, J. (2018). Hierarchical optimization for the efficient parametrization of ODE models. *Bioinformatics*, 34(24):4266–4273.

Park, C. H., Cho, S. Y., Ha, J. D., Jung, H., Kim, H. R., Lee, C. O., Jang, I.-Y., Chae, C. H., Lee, H. K., and Choi, S. U. (2016). Novel c-met inhibitor suppresses the growth of c-met-addicted gastric cancer cells. *BMC Cancer*.

Steiert, B., Timmer, J., and Kreutz, C. (2016). L1 regularization facilitates detection of cell type-specific parameters in dynamical systems. *Bioinformatics*, 32(17):i718–i726.
