# Supplementary material for: Model-based analysis of response and resistance factors of cetuximab treatment in gastric cancer cell lines
Source: PLoS Comput Biol. 2020 Mar 2;16(3):e1007147. doi: 10.1371/journal.pcbi.1007147 (PMC7067490; doi:10.1371/journal.pcbi.1007147)
Supplement: S1 Table — The following kinetic rates were identified as significantly different between the cell lines by the ANOVA statistical test. All kinetic rates belonging to the same module were simultaneously tested (not individually) resulting in 8 different model candidates. (PDF) [file pcbi.1007147.s014.pdf]

Module	Reaction	Description
EGFR turnover	R10: (pEGFR:EGF)$_2$ $\rightarrow$ (pEGFR:EGF)$_{2,\text{endocytic vesicle}}$	Endocytosis of pEGFR
	R14: (pEGFR:EGF)$_{2,\text{endocytic vesicle}}$ $\rightarrow \emptyset$	Lysosomal degradation of endocyted pEGFR
	R2: EGFR $\rightarrow \emptyset$<br>R5: EGFR:CET $\rightarrow \emptyset$<br>R12: EGFR:EGF $\rightarrow \emptyset$<br>R13: (EGFR:EGF)$_2$ $\rightarrow \emptyset$	Basal membrane turnover
	R11: (pEGFR:EGF)$_{2,\text{endocytic vesicle}}$ $\rightarrow$ EGFR + EGFR	EGFR recycling endosomes
RAS-MAPK signaling	R39: RAS-GDP $\rightarrow$ RAS-GTP	Basal activation of RAS
	R40: RAS-GDP + (pEGFR:EGF)$_2$ $\rightarrow$<br>$\rightarrow$ RAS-GTP + (pEGFR:EGF)$_2$<br>R41: RAS-GDP + (pEGFR:EGF)$_{2,\text{endocytic vesicle}}$ $\rightarrow$<br>$\rightarrow$ RAS-GTP + (pEGFR:EGF)$_{2,\text{endocytic vesicle}}$	EGFR dependent activation of RAS
	R46: RAS-GTP $\rightarrow$ RAS-GDP	Basal GTPase activity of RAS
	R47: ERK + RAS-GTP $\rightarrow$ pERK + RAS-GDP	RAS dependent activation of MAPK
PI3K-AKT signaling	R65: AKT + pPI3K $\rightarrow$ pAKT + pPI3K<br>R66: AKT + pMPI3K $\rightarrow$ pAKT + pMPI3K	PI3K and MPI3K dependent activation of AKT
	R67: pAKT $\rightarrow$ AKT	Basal dephosphorylation of pAKT
	R56: pPI3K $\rightarrow$ PI3K<br>R64: pMPI3K $\rightarrow$ MPI3K	Basal dephosphorylation of pPI3K and pMPI3K
