# Supplementary material for: Model-based analysis of response and resistance factors of cetuximab treatment in gastric cancer cell lines
Source: PLoS Comput Biol. 2020 Mar 2;16(3):e1007147. doi: 10.1371/journal.pcbi.1007147 (PMC7067490; doi:10.1371/journal.pcbi.1007147)
Supplement: S2 Table — Prior mean values were converted to 10-logarithmic scale and used for the parameterization of the model. Note that the same kinetic rate can appear in different reactions, e.g., receptor endocytosis in R10, R22 and R34. (PDF) [file pcbi.1007147.s015.pdf]

**Reaction**	**Value**	**Publication**
R6, R7: EGFR + EGF $\rightleftharpoons$ EGFR:EGF	$\log_{10}(2)$	Klein et al. [2004], page 1
R3, R4: EGFR + CET $\rightleftharpoons$ EGFR:CET	$\log_{10}(0.39)$	[Kim and Grothey, 2008], page 2
R10: $(\text{pEGFR:EGF})_2 \to (\text{pEGFR:EGF})_{2,\text{endocytic vesicle}}$<br>R22: $\text{pMMET}_2 \to \text{pMMET}_{2,\text{endocytic vesicle}}$<br>R34: pMMET:pEGFR $\to$ $\text{pMMET:pEGFR}_{\text{endocytic vesicle}}$	$\log_{10}(0.25)$	[Sorkin and Duex, 2010], page 19
R67: pAKT $\to \emptyset$	$\log_{10}(1.5 \times 60)$	[Schöberl et al., 2009], kinetic rate k_f83
